# Insulin-like growth factor 2 reduces Huntington’s disease aggregates via AKT and NF-κB signaling in huntington’s disease

**DOI:** 10.1186/s13578-025-01452-4

**Published:** 2025-07-26

**Authors:** Yun-Shiuan Tung, Chih-Wei Tung, Siew Chin Chan, Yi-Ching Chen, Po-Ming Wu, Pei-Hsun Cheng, Chuan-Mu Chen, Shang-Hsun Yang

**Affiliations:** 1 Department of Physiology, College of Medicine, National Cheng Kung University, Tainan, 70101 Taiwan; 2 Institute of Basic Medical Sciences, College of Medicine, National Cheng Kung University, Tainan, 70101 Taiwan; 3 Department of Pediatrics, College of Medicine, National Cheng Kung University Hospital, National Cheng Kung University, Tainan, 70101 Taiwan; 4 Department of Life Sciences, College of Life Sciences, National Chung Hsing University, Taichung, 40227 Taiwan

**Keywords:** Insulin-like growth factor 2(IGF2), Protein kinase B (AKT), Nuclear factor kappa-light-chain-enhancer of activated B cells(NF-κB), Extracellular vesicles, Mutant Huntingtin, Aggregates, Huntington’s disease

## Abstract

**Background:**

Aggregation of misfolded mutant Huntingtin (mHTT) is a pathological characteristic in Huntington’s disease (HD), implying clearance of mHTT is a therapeutical direction for this neurodegenerative disorder. Based on previous studies, Insulin-like growth factor 2 (IGF2) enhances microfilament polymerization in HD models; however, the role of IGF2 against mHTT aggregates is still unclear.

**Results:**

Here, we demonstrate that IGF2 expression is significantly lower in symptomatic HD patients compared to presymptomatic individuals, and IGF2 activation mechanistically enhances phosphorylation of Protein Kinase B(AKT; serine/threonine kinase), which subsequently reduces mHTT aggregates in vitro. Furthermore, IGF2 stimulates Nuclear factor kappa-light-chain-enhancer of activated B cells (NF-κB) signaling, promoting the secretion of mHTT within extracellular vesicles, thereby aiding cellular clearance. In vivo studies in R6/2 HD transgenic mice reveal that IGF2 administration improves motor functions and decreases mHTT levels.

**Conclusions:**

Collectively, our findings elucidate the multifaceted role of IGF2 in HD, highlighting its therapeutic potential through modulation of AKT and NF-κB signaling pathways.

**Supplementary Information:**

The online version contains supplementary material available at 10.1186/s13578-025-01452-4.

## Graphical Abstract

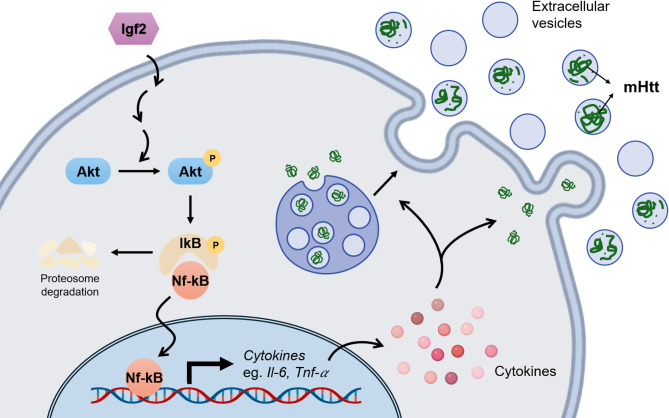

## Introduction

Huntington’s disease (HD), known as Huntington’s chorea, stands as a progressive neurodegenerative disease caused by mutations in the *Huntingtin* gene (m*HTT*) with expanded CAG repeats [1–3]. This is an autosomal dominant inherited disorder, and the symptoms would manifest depending on the amount of CAG repeats in *mHTT*. In cellular level, the expanded CAG repeats form the misfolding of mHTT, further leading to oxidative stress, mitochondrial dysfunctions, formation of aggregates, progressive neuron death, etc [4–8]. Finally, it causes neurodegeneration and clinical symptoms in patients. The presentation of HD encompasses motor activity deficits, cognitive impairment, and profound challenges in emotional regulation and personality [1, 2, 9]. Currently, it exists no cure for HD; therefore, understanding the mechanisms and pathogenesis of HD is crucial for developing effective therapies.

*Insulin-like growth factor 2* (*IGF2*) gene, a member of the *insulin-like growth factor* (*IGF*) family, is located on chromosome 11p15.5 in humans. IGF2 activates a specific receptor, insulin-like growth factor 2 receptor (IGF2R), which acts as a scavenger receptor to regulate the availability of IGF2 in the extracellular environment [10]. Furthermore, IGF2 plays an important role during embryonic development and adulthood, and distributes widely throughout the body, including in the liver, intestine, and even the brain [11, 12]. It dominantly functions towards mitochondria regulation [13], insulin-like metabolic responses [14], and the regulation of various physiological processes. In central nervous system (CNS), IGF2 and its receptor, IGF2R, are expressed at high levels, and have shown to involve in neuronal functions related to brain development, memory formation, cognition, plasticity, etc [15, 16]. In addition, IGF2 has shown the neuroprotective functions in several neurodegenerative diseases, such as Alzheimer’s disease [17], Parkinson’s disease [18], amyotrophic lateral sclerosis [19], and spinal muscular atrophy [20]. Moreover, our laboratory has shown IGF2 enhances actin dynamics and structures, neurite outgrowth and filopodia formation in HD models [9]. These results suggest understanding the regulatory mechanisms of IGF2 in CNS may offer insights into developing effective therapeutic interventions for different neurodegenerative diseases.

There are several IGF2 downstream signaling pathways, and the serine/threonine kinase (AKT, also known as PKB) signaling is one of the critical pathways, which functions to reduce the cell death and the apoptotic process, glucose uptake, angiogenesis, metabolism, etc [21, 22]. This AKT pathway has been reported to act as a neuroprotective mechanism by regulating the mammalian target of rapamycin (mTOR) and glycogen synthase kinase 3 beta (GSK3β) in brains [23–27], suggesting the critical roles of AKT signaling in neuroprotection. In addition to the AKT signaling, Nuclear factor kappa-light-chain-enhancer of activated B cells (NF-κB), a transcription factor, has also been reported to involve in the downstream of IGF2 signaling, and this transcription factor regulates immune and inflammatory responses, cell survival and proliferation, extrusion of extracellular vesicles, etc [27–29]. NF-κB typically locates in the cytoplasm and binds to an inhibitory protein, nuclear factor kappa-B (I-κB), in an inactive state. Upon activation, these I-κBs are phosphorylated and degraded by IκB kinase (IKK), leading to that NF-κB translocates into the nucleus to modulate the transcription of target genes involved in various cellular processes [29]. Moreover, NF-κB has been reported to provide anti-apoptotic functions and lead to neuroprotection [1, 28]. These results highly suggest IGF2 may work through above cooperative pathways to achieve neuroprotective functions.

Based on our previous studies, we have shown IGF2 is beneficial for actin dynamics and filopodia formation, which enhances neurite outgrowth, through Cdc42 activation in HD models [9]. Due to that IGF2 offers neuroprotective functions and the mHTT aggregates are important neuropathological characteristics in HD, we are curious about the effects of IGF2 on mHTT aggregates and related mechanisms. Here, we demonstrate the regulatory functions of IGF2 in mHTT aggregates in vitro and in vivo, and expect to provide a potential direction for the development of therapy in HD.

## Materials and methods

### Bioinformatic analysis (GEO)

The expression profiling of IGF2 in HD patients and controls was analyzed using data from the GEO database (https://www.ncbi.nlm.nih.gov/geo/). The datasets utilized in this study are accessible under the accession number GSE1767 [30], GSE97100 [31] and GSE74201 [32, 33].

### Cell culture

Cell studies were performed using Neuro-2a neuroblastoma cells (N2a; ATCC, CCL-131) and 293FT cells (Invitrogen, R70007). N2a cells were cultured in Eagle’s Minimum Essential Medium (Gibco, 41500-034), supplemented with 10% fetal bovine serum (FBS, Hyclone) and 1 mM sodium pyruvate, maintained at 37 °C in a humidified incubator with 5% CO2. 293FT cells were grown in Dulbecco’s Modified Eagle’s Medium (Gibco, 12800-017) supplemented with 10% FBS and L-glutamine, under the same conditions. Cells were passaged every 2–3 days upon reaching 80–90% confluency. For the BAY 11-7082(Sigma, 19542-67-7) treatment, N2a cells were pretreated with 5µM BAY 11-7082 for 1 h before the transfection. DMSO was used as the control.

### Plasmids and transfection

The constructs utilized in this study include mHTT, mHTT-GFP, IGF2, GIGF2, shAKT, pNF-kB-Luc, and β-gal. The mHTT construct comprises the exon 1 region of the human *HTT* gene containing 84 CAG repeats, driven by a human ubiquitin promoter. mHTT-GFP consists of the *mHTT* sequence fused to enhanced green fluorescent protein (EGFP), also under the control of a human ubiquitin promoter. The IGF2 construct contains a FLAG tag appended to the coding sequence of human IGF2 mRNA (accession number: NM_000612.6), also driven by a human ubiquitin promoter. GIGF2 encodes a fusion protein of EGFP and IGF2 under the regulation of a cytomegalovirus (CMV) immediate early promoter. The shAKT (TRCN0000304684) and control shRNA (Scramble, ASN0000000003) constructs were sourced from the National RNAi Core Facility at Academia Sinica, Taiwan. The pNF-kB-Luc plasmid (Clontech) contains the firefly *luciferase* gene under the regulation of the herpes simplex virus thymidine kinase promoter coupled with the NF-κB-binding sequence (GCCCTTAAAG). The β-gal reporter contains the coding sequence of *β-galactosidase* driven by a CMV promoter. All plasmids were transfected into cells via Lipofectamine™ 3000 Transfection Reagent (Invitrogen, L3000015). After 48 h of incubation, the cells were washed with PBS and harvested for subsequent analyses.

### Western blotting (WB) analysis

Cells or tissues were lysed using RIPA buffer containing 50 mM Tris (pH 8.0), 150 mM NaCl, 1 mM EDTA (pH 8.0), 1 mM EGTA (pH 8.0), 0.1% Sodium dodecyl sulfate (SDS), 0.5% deoxycholate, and 1% Triton X-100, supplemented with protease inhibitor (ChemCruz, sc-29131), followed by sonication to extract raw proteins. Protein concentration was measured via the Coomassie (Bradford) protein assay kit (Thermo Fisher Scientific, 23200) to ensure equal amounts of samples. Samples were subjected for SDS polyacrylamide gel electrophoresis (SDS-PAGE), and then transferred to polyvinylidene difluoride (PVDF) membrane. The membrane was blocked with 5% skimmed milk before hybridizing with specific primary and secondary antibodies. The primary antibodies included EM48 (Merck Millipore, MAB5374, 1: 1000), γ-tubulin (Sigma, T6557, 1:10000), IGF2 (Abcam, ab9574, 1: 1000), phospho-Akt (Ser473) (Cell signaling, 4060 S, 1:1000), AKT (Cell signaling, 9272 S, 1:1000), I-κB (Cell signaling, 1:1000), NF-κB (Cell signaling, 4764 S, 1:1000), phosphor-NF-κB(Cell Signaling, 3033, 1:1000), GAPDH (GeneTex, GTX100118, 1:6000) and HA-Taq (Roche, 1:1000, 11867423001). Secondary antibodies conjugated with peroxidase (KPL) were used for detection of the primary antibodies. Protein expression levels were detected by T-Pro LumiLong Plus Chemiluminescent Substrate Kit (T-Pro Biotechnology, JT96-K004M) using an imaging system (Wealtec Corp.) and the results were quantified using ImageJ software (NIH) for statistical analysis.

### Nuclear and Cytosolic Fractionation

Transfected N2a cells were separated into nuclear and cytosolic fractions as described previously with modification [4, 28]. Briefly, cells were lysed in cytoplasmic extraction buffer (CEB, 10 mM HEPES pH 7.9, 10 mM KCl, 0.1 mM EDTA, 0.3% NP-40), and a portion of the lysate was retained as the whole-cell lysate. The nuclear fraction was obtained by centrifuging the lysate at 16,000 × g for 10 min at 4 °C. Afterward, the supernatant, containing the cytosolic fraction, was carefully collected. The nuclear pellet was washed twice with CEB to eliminate any remaining cytosolic components, followed by lysis in RIPA buffer. All fractions were then subjected to ultrasonication for WB analysis.

### Promoter-reporter assay

N2a cells were co-transfected mHTT, pNF-kB-Luc and β-gal reporter constructs with either IGF2 or an empty vector for 48 h. The β-gal expression was used as an internal control. The promoter-reporter assay was carried out according to the manufacturer’s protocol(Promega). Luciferase activity was measured using a luminometer with a 2-second delay followed by a 10-second reading (absorbance: 420 nm). The luciferase activity was normalized to β-gal expression to account for variations in transfection efficiency.

### Real-time quantitative PCR

Total RNA was isolated from transfected cells using TRIzol^®^ Reagent (Invitrogen, 15596026), according to the manufacturer’s instructions. The RNA concentration was determined using a UV spectrophotometer (Merinton). To eliminate any residual DNA, total RNA was treated with DNase I (New England Biolabs, M0303S), followed by reverse transcription to synthesize complementary DNA for subsequent real-time PCR analysis. Real-time PCR was conducted using SYBR^®^ Green PCR Master Mix (Applied Biosystems, 4309155). The expression levels of mHTT, TNF-α and IL-6 mRNA were quantified using specific primer pairs for mHTT (forward: 5’- ATGGCGACCCTGGAAAAGCT-3’, reverse: 5’- TGCTGCTGGAAGGACTTGAG-3’), TNF-α (forward: 5’-GTCTCAGCCTCTTCTCATTCCT-3’, reverse: 5’-TGTGAGTGTGAGGGTCTGGG-3’) and IL-6 (forward: 5’-TCTTGGGACTGATGCTGGTGA-3’, reverse: 5’-TGCACAACTCTTTTCTCATTTCC-3’). 18 S rRNA primers (forward: 5’-CGGCTACCACATCCAAGGAA-3’, reverse: 5’-CCTGTATTGTTATTTTTCGTCACTACCT-3’) and β-Actin primers (forward: 5’-ggctgtattcccctccatcg-3’, reverse: 5’-tacctctcttgctctgggcc-3’) were used as an internal control for normalization. Data acquisition and analysis were performed using StepOne software v2.1 (Applied Biosystems).

### Extracellular vesicles isolation

N2a cells were transfected with different constructs, and the medium was replaced with serum-free OPTI-MEM on the second day. The cells were then cultured for an additional 2 days. Conditioned media from the transfected cells were collected and centrifuged at 2,000 x g for 30 min at 4 °C to remove cell debris. The supernatant was then concentrated up to 15-fold using Microsep Advance Centrifugal Devices with Omega Membrane 100 K (Pall Corporation, MAP100C41). All concentrated media were then subjected to ultrasonication for subsequent WB analysis.

### Transgenic mice

R6/2 mice transgenic mice, which express exon 1 of the *HTT* gene under the control of the human *HTT* promoter, were used in this study. All experimental protocols were approved by the Institutional Animal Care and Use Committee at National Cheng Kung University, Taiwan. Genotyping via polymerase chain reaction (PCR) was performed using PCR primers, including 5’-GGCGACCCTGGAAAAGCTGA-3’ and 5’-TGAGGAAGCTGAGGAGGCGG-3’. The presence of the transgene was confirmed by the detection of a 490 bp amplicon. Animals were housed under a 14:10 h light-dark cycle with *ad libitum* access to chow and water. Both male and female mice were included in this study.

### Lentivirus production

Lentivirus expressing GIGF2 was produced following the method described in our previous studies [9, 34]. In brief, 293FT cells were co-transfected with the GIGF2 plasmid, packaging plasmid (pCMV-delta 8.9), and envelope plasmid (pVSV.G) using calcium phosphate transfection. After 48 h, the lentivirus-containing medium was harvested and concentrated to generate high titer viruses. The concentrated viral pellet was re-suspended in PBS and stored at -80 °C for future experiments.

### Stereotactic injection

The injection procedure was approved by the Institutional Animal Care and Use Committee (IACUC) under protocol number 111,063. Prior to stereotactic injection, six-week-old mice were anesthetized with a combination of Zoletil 50 (50–60 mg/kg) and Xylazine (2.3–2.7 mg/kg) intraperitoneally. Once anesthetized, the mice were positioned in the stereotaxic frame, and ensured that their teeth were secured and their ears were fixed to maintain a horizontal position. The fur on the head was shaved, and the scalp was incised to expose the skull using a spreader. The bregma and lambda positions were identified, and StereoDrive software (Neurostar) was used to map these landmarks. Lentivirus was injected into the striatum using the following stereotaxic coordinates: +0.38 mm anterior-posterior, ± 1.85 mm mediolateral, and + 3.57 mm dorsal-ventral. Two 1 mm-wide holes were drilled at the identified locations, and 2 µL of the lentivirus suspension was injected over a period of 5 min, with the needle remaining in place for an additional 5 min post-injection. After the procedure, the wound was treated with betadine and sutured using absorbable sutures. The mice were placed on a 37 °C warming pad to recover from anesthesia and monitored for 3 days postoperatively to ensure full recovery and the absence of any adverse effects.

### Behavioral tests

#### Rotarod

Motor coordination and balance were assessed using an accelerated rotarod apparatus (Singa). The experiment consisted of training and testing phases. During the training phase, each mouse was placed on the rotarod at a constant speed of 5 rpm for up to 3 min per session, repeated three times with 3-minute intervals between sessions. In the testing phase, mice were subjected to an acceleration from 4 to 40 rpm over a 5-minute period, and the latency to fall was recorded. Testing included three trials per mouse, with a 10-minute recovery period between each trial.

#### Grip strength

Muscular strength was assessed using a grip strength meter (Bioseb), which automatically recorded the force required for the mouse to release its grip. During the test, each mouse was placed on a metal grid and gently held by the tail. The assessment was conducted in a single session consisting of three consecutive trials, and the final result was determined as the average force from these trials.

### Immunofluorescence staining

Mouse brains were harvested following PBS perfusion and fixed in 4% paraformaldehyde for one week. The brains were then immersed in 30% sucrose with 0.1 M phosphate buffer for an additional week before being cryosectioned into 25 μm slices. The sections were blocked using a blocking buffer, primary antibodies were then applied and the slides were incubated at 4 °C overnight. Immunofluorescence staining analysis was performed with antibodies against the EM48 (Merck Millipore, MAB5374, 1:1000). Subsequently, the brain sections were rinsed with PBS and incubated for one hour at room temperature in highly cross-absorbed fluorescent-conjugated secondary antibodies Alexa Fluor^®^ 594 goat anti-mouse IgG (Invitrogen, A-11032, 1:1000). Nuclei were stained using Hoechst 33,342 (Invitrogen, H3570). Afterward, the brain sections were rinsed with PBS, air-dried, cover slipped, and mounted onto slides. The fluorescent signals were captured by DMi8 fluorescent microscope (Leica) and analyzed with Metamorph software.

### Statistical analysis

The data are presented as means ± standard deviation (SD). An unpaired one-tailed t-test was performed to assess differences between the two groups. All analyses were conducted using GraphPad Prism 5 software. The levels of significance are denoted as follows: *P < 0.05, **P < 0.01, and ***P < 0.001.

## Results

### IGF2 suppresses the mHTT aggregates through activation of p-AKT

**Fig. 1:**
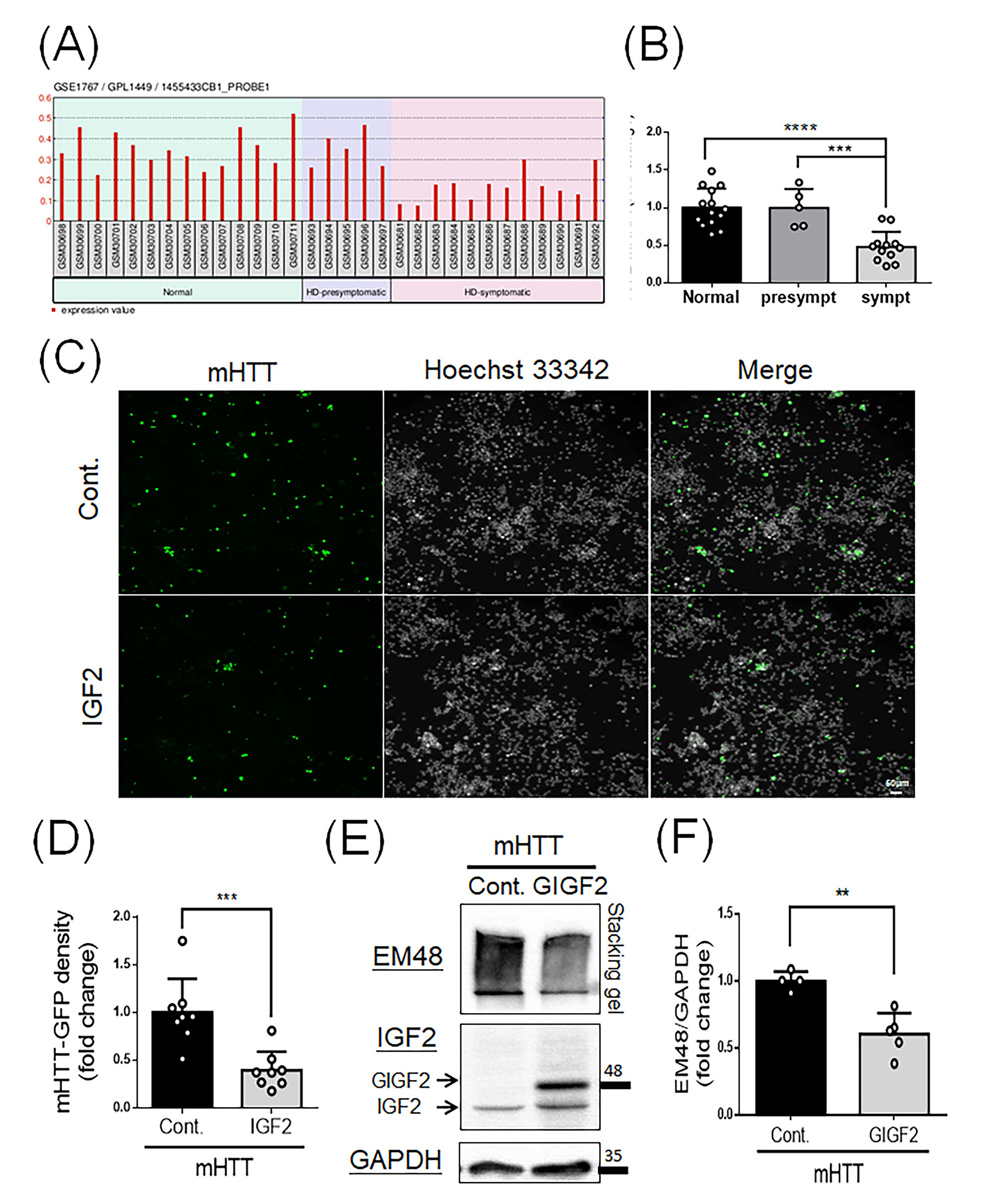
IGF2 suppresses the mHTT aggregates in N2a cells. **(A** and **B)** IGF2 gene expression in peripheral blood samples from healthy individuals (*N* = 14), HD-presymptomatic patients(*N* = 5) and HD-symptomatic patients(*N* = 12) are obtained from the GEO database (GSE1767). **(A)** Raw data of IGF2 mRNA expression in peripheral blood samples. **(B)** Quantitative analysis from (A) shows a significant decrease of IGF2 in HD-symptomatic patients compared to the other two groups. **(C-F)** N2a cells were transfected mHTT, G84Q, with control(Cont.) empty vector or IGF2/GIGF2 for 48 h, and then subjected for observing fluorescent signals of mHTT or Western blotting. **(C)** Fluorescent images show the expression of mHTT(green) after transfection of control or IGF2. Nuclei were stained with Hoechst 33,342(white). Scale bar: 50 μm. **(D)** Quantification of mHTT from(C) shows a significant decrease in the mHTT in the IGF2 group. **(E)** Western blotting shows the expression of mHTT after transfection of control(Cont.) empty vector and GIGF2 (IGF2 fusion with GFP). **(F)** Quantitative analysis from (E) shows a decrease of mHTT expression after the GIGF2 treatment. Values represent the mean ± SD. **p < 0.01. ***p < 0.001. ****P < 0.0001

Our previous studies have shown IGF2 enhances neurite outgrowth through Cdc42 activation in HD models [9], highly suggesting IGF2 may play a neuroprotective role in HD. To further demonstrate the working mechanism of IGF2 in HD, we first are curious about the expression profiles of IGF2 in HD patients during the disease progression. We analyzed Gene Expression Omnibus (GEO) database, GSE1767, using peripheral blood samples from healthy individuals, HD-presymptomatic patients and HD-symptomatic patients to access the IGF2 expression profiling. The IGF2 expression profiling are extracted from GSE1767 dataset (Fig. 1A), and quantitative results show that there is no difference between HD-presymptomatic patients and healthy individuals (Fig. 1B). Interestingly, the expression of IGF2 is significantly lower in HD-symptomatic patients compared to those of HD-presymptomatic patients and healthy individuals (Fig. 1B), which has been partly reported in our previous study [9]. To further support the importance of IGF2 in HD via GEO database, we surveyed the other two datasets, GSE97100 using HD patient iPSC-derived brain microvascular endothelial cells(Supplementary Fig. 1A and 1B) [31] and GSE74201 using HD patient iPSC-derived differentiated neural stem cells(Supplementary Fig. 1C and 1D) [32, 33]. We also found IGF2 expression is lower in HD groups(Supplementary Fig. 1), suggesting IGF2 may be related to neuropathological characteristics in HD patients.

In order to explore the advanced effects of IGF2 on the one critical characteristic, mHTT aggregate, in HD, we delivered the *G84Q*, used as *mHTT*, and *IGF2* constructs into the N2a neuroblastoma cells, and examined the profiling of mHTT aggregates. We first examined the transcription level of mHTT after IGF2 overexpression. Through the analysis of quantitative PCR(Q-PCR), mRNA level of mHTT is increased in the IGF2 group compared to that of the control group(Supplementary Fig. 2). Due to mHTT fused with GFP, we observe the GFP signals to indicate the mHTT aggregates. As shown in the Fig. 1C and D, the mHTT aggregates decrease after the IGF2 transfection (Fig. 1C, bottom panel) compared to that of the empty vector control (Fig. 1C, top panel; Fig. 1D). We also designed an *IGF2* construct fused with *GFP* gene to form the *GIGF2* plasmid because this design could facilitate the observation of IGF2 expression in vitro and in vivo. To examine the effects of *GIGF2* on mHTT aggregates, GIGF2 and G84Q were contransfected into N2a cells, and mHTT aggregates were detected via Western blotting. As shown in the Fig. 1E, higher expression level of exogenous GIGF2 is observed, leading to significantly lower expression of mHTT aggregates detected using an EM48 antibody (Fig. 1F). These results suggest IGF2 could suppress mHTT aggregates in vitro.

**Fig. 2:**
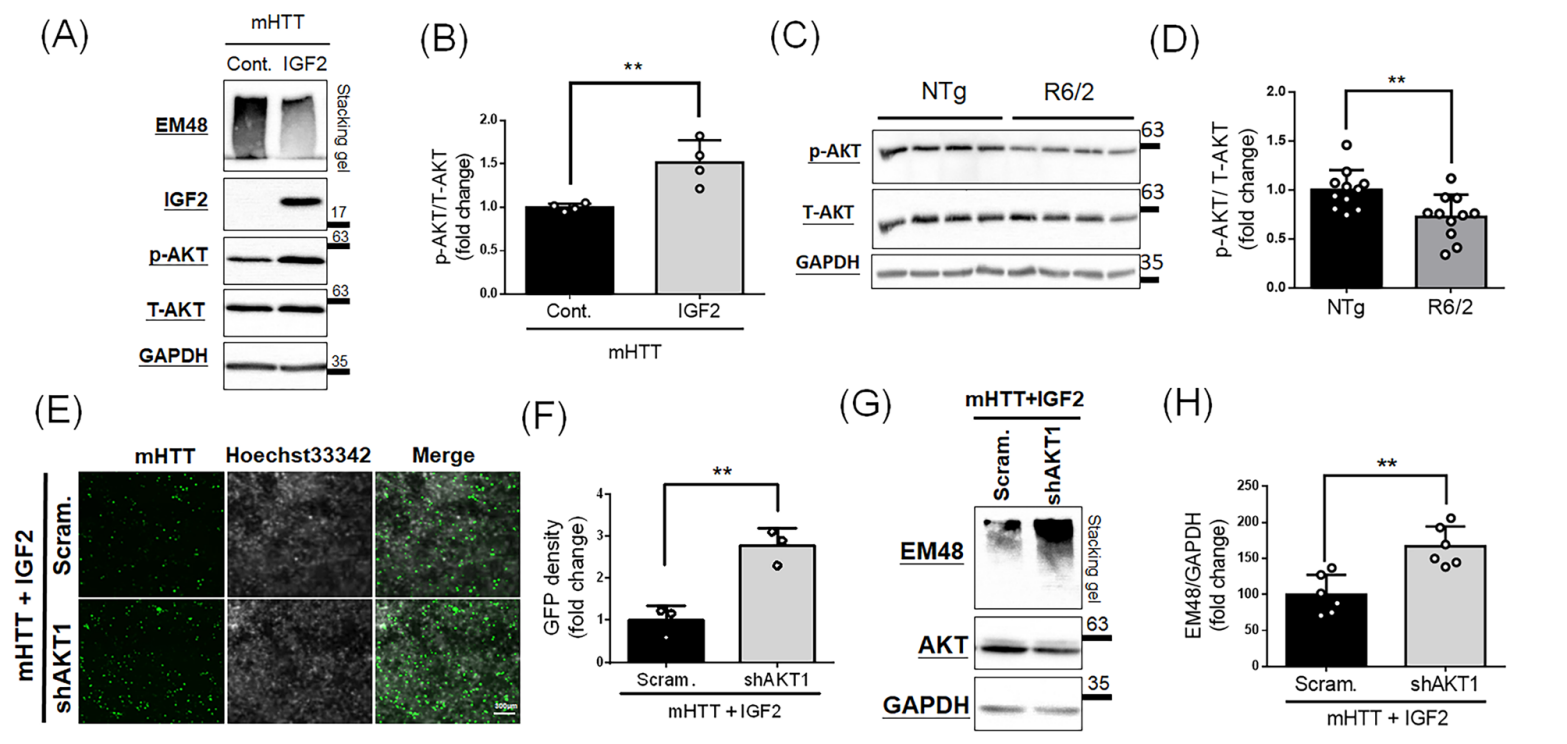
IGF2 suppresses mHTT aggregates through activation of p-AKT. **(A and B)** N2a cells were transfected mHTT with control(Cont.) or IGF2 vectors for 48 h, and then subjected for Western blotting. **(A)** Western blotting shows the phosphorylation level of AKT after IGF2 overexpression. Phospho-AKT (Ser473) and total AKT antibodies were used to detect phosphorylation level. GAPDH serves as an internal control. **(B)** Quantitative analysis from **(A)** shows a increase of p-AKT/T-AKT level after the IGF2 treatment. **(C and D)** Cortex samples from non-transgenic (NTg) and R6/2 transgenic mice at 1 month of age. **(C)** Western blotting shows phosphorylation level of AKT in NTg and R6/2 HD transgenic mice. **(D)** Quantitative analysis from **(C)** shows the decrease of p-AKT/T-AKT level in R6/2 transgenic mice. Values represent the mean ± SD. **(E-H)** N2a cells were transfected mHTT and IGF2 with scramble shRNA(Scram.) or shAKT1 for 48 h, and then subjected for observing fluorescent signals of mHTT or Western blotting. **(E)** Fluorescent images show the expression of mHTT(green) after transfection of different constructs as indicated in the left side. Nuclei were stained with Hoechst 33,342(white). Scale bar: 300 μm. **(F)** Quantification of mHTT from **(E)** shows a significant increase of mHTT signals after knockdown of AKT. **(G)** Western blotting shows the expression of mHTT after transfection of different constructs. **(H)** Quantitative analysis from **(G)** shows a significant increase of mHTT aggregates after knockdown of AKT. Values represent the mean ± SD. **p < 0.01

IGF2 has been reported to activate phosphorylation of AKT to initiate downstream functions in several tissues [21, 22]. Since we observe IGF2 suppresses mHTT in HD cells, we are curious about whether IGF2 could function through the AKT signaling. We examined the expression profiling of phosphor-AKT (Ser473; p-AKT) and total AKT (T-AKT) in N2a cells transfected with *G84Q* and *IGF2*. As shown in Fig. 2A, overexpression of IGF2 does suppress the mHTT aggregates, and increases the p-AKT/T-AKT ratio as well (Fig. 2B). Furthermore, we also explored the profiling of p-AKT and T-AKT in brains of R6/2 HD transgenic mice, which carry exon 1 of *mHTT* transgene with approximate 150 CAG repeats driven by a HTT promoter [4, 35], at one month of age in vivo using Western blotting (Fig. 2C), showing the p-AKT/T-AKT ratio is significantly decreased in R6/2 HD transgenic mice (Fig. 2D). This result imply HD transgenic mice may not activate AKT signaling sufficiently to initiate the downstream functions.

Since the IGF2 could activate phosphorylation of AKT and suppress mHTT aggregates in HD cells, the role of AKT signaling involving in IGF2-suppressed mHTT aggregates is further demonstrated. To achieve this purpose, short hairpin RNA(shRNA) targeting AKT1 (shAKT1) or scramble shRNA were cotransfected with *G84Q* and *IGF2* into N2a cells, and these transfected cells were then subjected for observing fluorescent signals of mHTT (Fig. 2E) and Western blotting (Fig. 2G). In the observation of mHTT signals, as AKT is knocked down by shAKT1 in N2a cells overexpressing *mHTT* and *IGF2* (Fig. 2E, bottom panel), the mHTT(GFP) signaling is increased compared to that of scramble shRNA(Fig. 2E, top panel; Fig. 2F). Upon the Western blotting (Fig. 2G), the expression level of AKT is decreased by shAKT1, and the mHTT aggregates are increased simultaneously in the stacking gel (Fig. 2G and H). These results highly suggest AKT signaling has critically involved in the beneficial effects of IGF2 on reduction of mHTT aggregates.

### IGF2 activates NF-κB signaling to increase mHTT in extracellular vesicles secreted in culture medium

Based on above results, we confirm IGF2 triggers AKT signaling to reduce mHTT; however, it remains unknown that how to link AKT signaling to mHTT reduction. One downstream component of the AKT signaling pathways is NF-κB, which upregulates inflammatory factors and induces inflammation [36]. In addition, NF-κB activation has been reported to stimulate the production of extracellular vesicles (EVs) [37, 38], which may play a role in clearance of cytosol proteins [37, 38]. Accordingly, we hypothesize IGF2 may trigger AKT signaling, then activate NF-κB transcription and further enhance EVs production to reduce cytosolic mHTT.

**Fig. 3:**
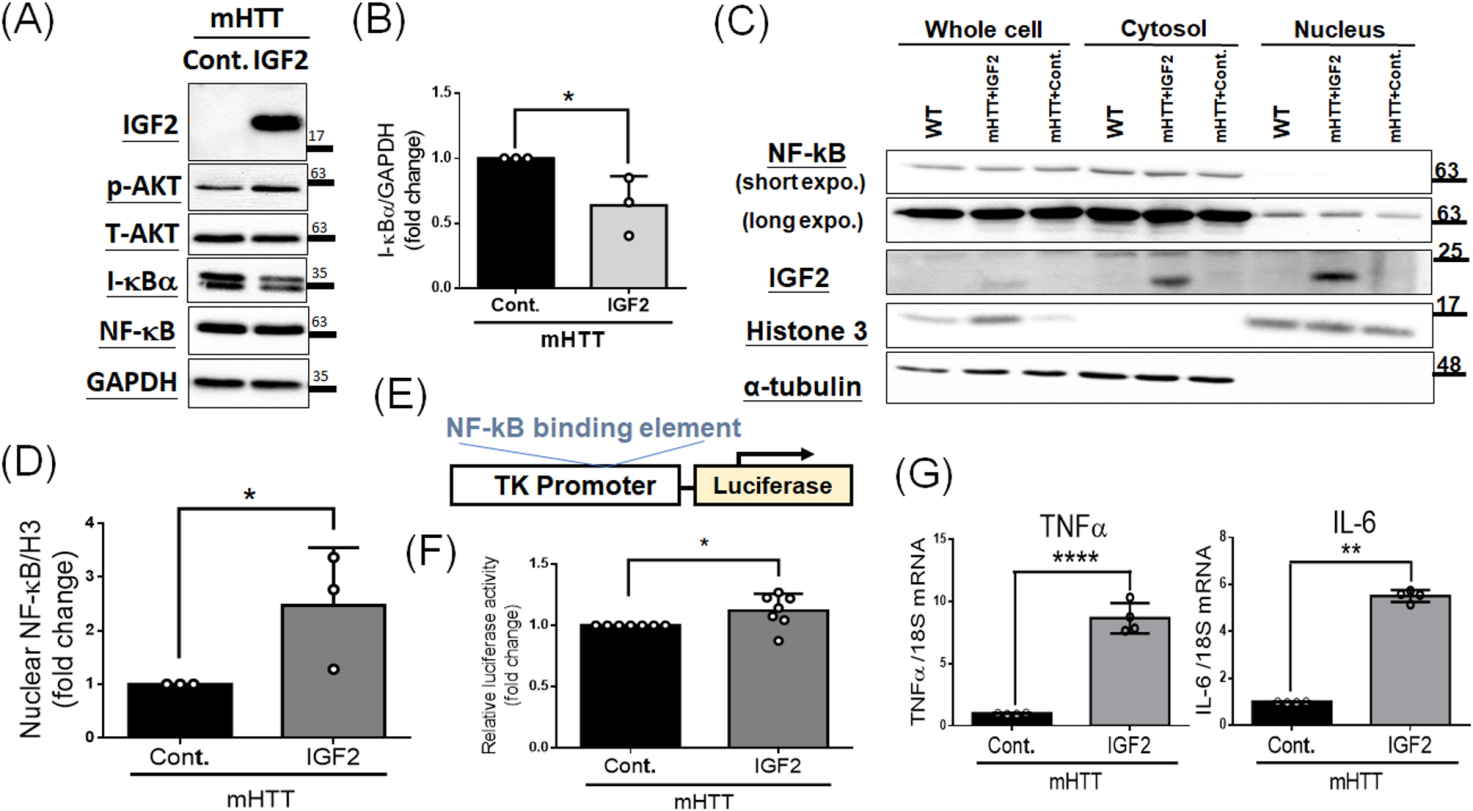
Overexpression of IGF2 activates NF-κB signaling in HD cells. **(A-D**,** G)** N2a cells were transfected mHTT with control(Cont.) or IGF2 vectors for 48 h, and then subjected for Western blotting and nuclear and cytoplasmic fractionation. **(A)** Western blotting shows the expression of IGF2, p-AKT, AKT, I-κBα and NF-kB after IGF2 overexpression. GAPDH serves as an internal control. **(B)** Quantitative analysis from **(A)** shows a decrease of I-κBα level after the IGF2 treatment. **(C)** Western blotting from nuclear and cytoplasmic fractionation shows the expression of NF-kB after IGF2 overexpression. Histone 3 (H3) and α-tubulin were used as indicators for nuclear and cytosolic fractions, respectively. **(D)** Quantitative analysis from **(C)** shows a increase of NF-kB translocation into nucleus after the IGF2 treatment in HD cells. **(E** and **F)** N2a cells were transfected mHTT and a reporter construct carrying NF-kB binding elements in the promoter region and luciferase gene **(E)** with control(Cont.) or IGF2 vectors for 48 h, and then subjected for the promoter-reporter assay. **(F)** Results show that IGF2 increases luciferase activity in HD cells. **(G)** Real-time quantitative PCR shows IGF2 increases TNFα (Left panel) and IL-6 (Right panel) mRNA expression in HD cells. Data represent the mean ± SD. *p < 0.05. **p < 0.01. ****p < 0.0001

To examine this hypothesis, we transfected *G84Q* with control or IGF2 vectors into N2a cells, and analyzed the expression of I-kBα, an inhibitory protein binding to NF-kB, and NF-kB. As shown in the Fig. 3A, IGF2 does increase the p-AKT/T-AKT ratio. Moreover, IGF2 also reduces the I-kBα level (Fig. 3B), but does not affect the expression of NF-κB (Fig. 3A). Since translocation of NF-κB into nucleus is a critical step to activate NF-κB signaling [28], we further performed the cell nuclear and cytoplasmic fractionation using transfected cells from the Fig. 3A, and detected the protein level of NF-κB in two different fractions. Based on the Western blotting using different fractionations (Fig. 3C), proteins from cytosol or nucleus are separated well according to the indication of Histone 3 (a nucleus marker) and α-tubulin (a cytosolic marker), respectively. In the nucleus fraction, IGF2 significantly increases the expression of NF-κB(Fig. 3C, lane 8; Fig. 3D) compared to that of control (Fig. 3C, lane 9; Fig. 3D). This result suggests IGF2 enhances the NF-κB translocation into nucleus in HD cells. Furthermore, since NF-κB is a transcription factor, which could bind to promoter regions of downstream genes to activate gene transcription [28], we used a promoter reporter assay to examine the transcriptional activity of NF-κB. The Fig. 3E shows the reporter construct carrying a thymidine kinase promoter with the NF-kB-binding sequence in the 5’ region of a luciferase reporter gene [28]. After transfecting this reporter construct with *G84Q* and *IGF2* into N2a cells, we observe IGF2 significantly increases the luciferase activity compared to that of the control in HD cells (Fig. 3F). We further used real-time quantitative PCR to examine the mRNA expression of two NF-κB downstream genes, TNF-αand IL-6, using samples from Fig. 3A. Results show that IGF2 increases TNFα (Fig. 3G left panel) and IL-6 (Fig. 3G right panel) mRNA expression in HD cells, suggesting IGF2 activates NF-κB pathway in HD cells. Furthermore, since we have shown the importance the AKT signaling involving in IGF2 functions in HD, we also tried to knock down the AKT and detected the expression of I-kBα via Western blotting after overexpression of *G84Q* and *IGF2* (Supplementary Fig. 3A). As shown in Supplementary Fig. 3A and 3B, shAKT abolishes the decrease of I-kBα observed in Fig. 3A and B. We also further used these transfected cells to perform the cell nuclear and cytoplasmic fractionation, showing IGF2 could not enhance the translocation of NF-κB into nucleus in HD cells under the shAKT condition (Supplementary Fig. 3C, lane 5 and lane 6). Moreover, we further used BAY 11-7082, an NF-κB Inhibitor, to suppress the NF-κB activity in HD cells after IGF2 treatment, and examined the mHTT aggregates via Western blotting. As shown in the Supplementary Fig. 4, IGF2 did suppress the mHTT aggregates. However, as we pretreated these cells with BAY 11-7082 before transfection, we not only suppress the phosphor-NF-κB(pNF-κB) levels but also reverse the suppression of mHTT aggregates induced by IGF2(Supplementary Fig. 4). Taken above results together, not only IGF2 promotes NF-κB signaling in HD cells, but also the downstream AKT signaling of IGF2 involves in this NF-κB activation.

**Fig. 4:**
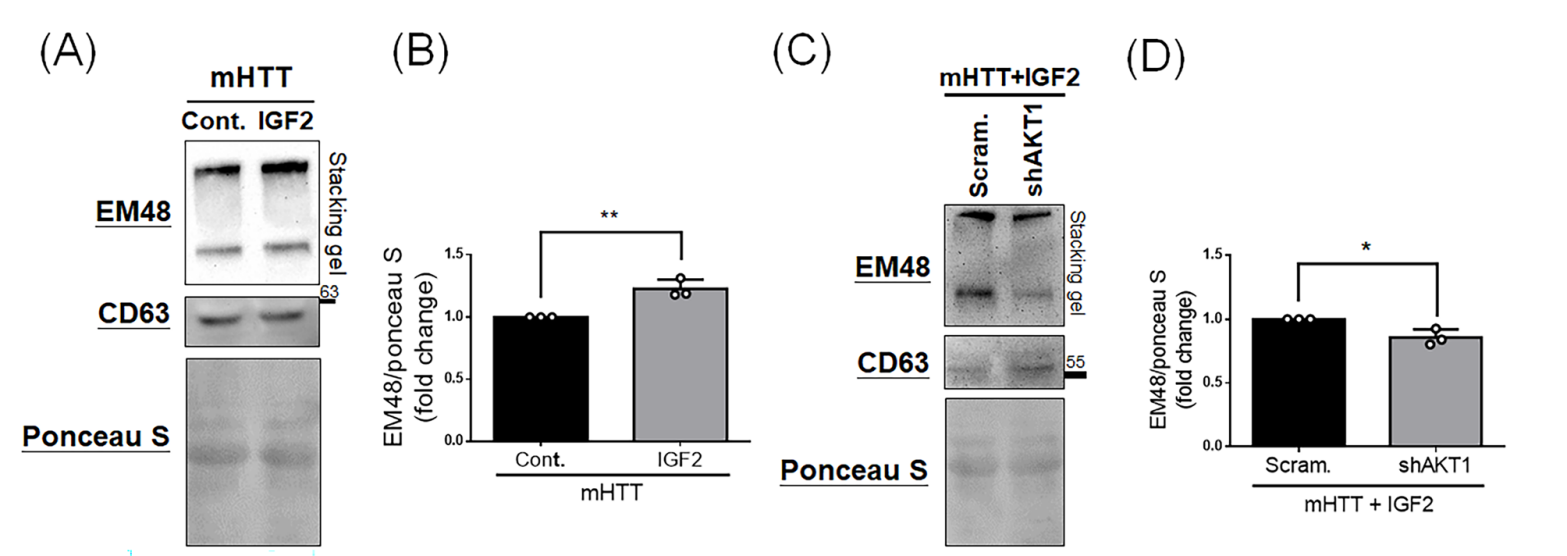
Knockdown of AKT reduces IGF2-mediated secretion of mHTT via EVs in HD cells. N2a cells were transfected with different constructs for 24 h, and the culture medium was replaced with Opti-MEM media. After 48-hour culture, supernatants were used to extract EVs, and then subjected for Western blotting. **(A)** Western blotting shows the expression of mHTT (indicated by EM48) and EVs (indicated by CD63) in culture media after cotransfection of mHTT with control(Cont.) or IGF2 into N2a cells. Ponceau S serves as an internal control. **(B)** Quantitative analysis from **(A)** shows an increase of mHTT in EVs after the IGF2 treatment. **(C)** Western blotting shows the expression of mHTT (indicated by EM48) and EVs (indicated by CD63) in culture medium of N2a cells transfected with mHTT and IGF2 as AKT1 was knocked down. Scramble shRNA(Scram.) is used as a control. Ponceau S serves as an internal control. **(D)** Quantitative analysis from **(C)** shows a decrease of mHTT in EVs after the knockdown of AKT1. Data represent the mean ± SD. *p < 0.05. **p < 0.01

Next, since NF-κB activation may enhance the EV production, which may assist to extrude the cytosolic proteins within EVs, we further examine the content of mHTT in EVs of culture medium. We extracted EVs from culture medium of N2a cells transfected *G84Q* with *IGF2* or control vectors, and then subjected EVs for Western blotting (Fig. 4A). The EVs are extracted successfully, indicated by the expression of CD63 (Fig. 4A), and mHTTs are detected using an EM48 antibody. Importantly, significantly higher mHTT level is detected under the IGF2 overexpression (Fig. 4B), suggesting IGF2 increases the EVs containing mHTT in culture medium. We also explore whether the AKT signaling involves in this IGF2-induced EVs in HD cells. We knocked down the AKT using shAKT1 in N2a cells transfected with *G84Q* and *IGF2*, extracted EVs from the culture medium of these transfected cells, and then performed the Western blotting. As shown in Fig. 4C and D, we observe significantly lower mHTT in the EVs of shAKT1 group compared to that of the scramble shRNA group. These results suggest IGF2 facilitates mHTT secretion within EVs in culture medium, and AKT signaling also involves in this IGF2-induced EV mechanism.

### IGF2 improves motor functions and reduces neuropathological mHTT in R6/2 HD Transgenic mice in vivo

**Fig. 5:**
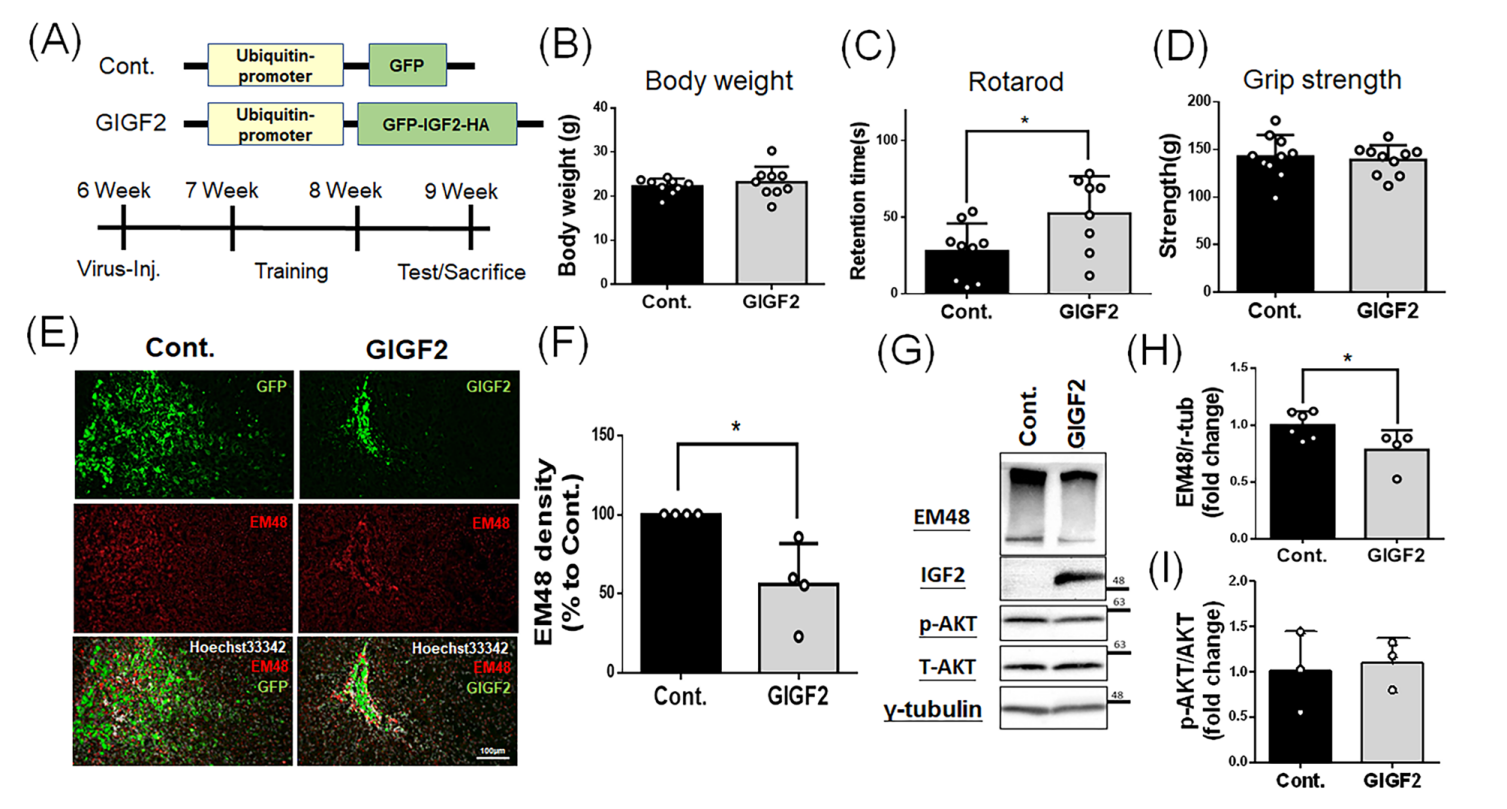
IGF2 improves motor functions and reduces neuropathological mHTT in R6/2 transgenic mice in vivo. **(A)** Schematic diagram illustrates the experimental procedure. Six-week-old R6/2 mice were subjected for bilateral stereotaxic viral injections using control (Cont.; GFP) or GIGF2 (IGF2 fused with GFP and HA-tag) lentiviruses. Injected mice were trained for behavioral tests at 7–8 weeks of age, and then performed behavioral tests at 9 weeks of age, further subjecting for neuropathological examinations. **(B)** Quantitative analysis shows there is no difference for the body weight between these two groups. **(C)** Quantitative analysis shows the R6/2 mice injected with GIGF2 perform significantly better rotarod behavior. **(D)** Quantitative analysis shows there is no difference for the grip strength between these two groups. **(E)** Immunofluorescent staining shows the expression profiling of mHTT (EM48; red) around the lentiviral expression sites (GFP or GIGF2; green) in striatum. Nuclei were stained with Hoechst 33,342(white). Scale bar: 100 μm. **(F)** Quantitative analysis from **(E)** shows the R6/2 mice injected with GIGF2 reduce mHTT aggregates compared to those of control. **(G)** Western blotting shows the expression profiling of EM48, IGF2, p-AKT and AKT in striatal tissues. Γ-tubulin serves as an internal control. **(H)** Quantitative analysis from **(G)** shows a decrease of mHTT in R6/2 mice injected with GIGF2 lentiviruses. **(I)** Quantitative analysis from (G) shows there is no difference for p-AKT/AKT ratio between these two groups. The data represent the mean ± SD. *p < 0.05

Since we have shown the critical role of AKT signaling in IGF2-induced neuroprotection in HD cells in vitro, we further demonstrate the neuroprotective effects of IGF2 in HD transgenic mice in vivo using stereotactical injection into striatum. Here, we used R6/2 HD transgenic mice displaying rapid disease onset and progression with symptoms typically appearing around 6–8 weeks of age. At 12 weeks of age, R6/2 HD transgenic mice show severe symptoms and generally survive only up to 13–15 weeks [39, 40]. To perform stereotactical injection, two types of lentiviruses, including control and GIGF2(Fig. 5A, top panel), were used to bilaterally inject into striatum of R6/2 HD transgenic mice at 6 weeks of age. The injected mice were trained for behavioral tests during 7–8 weeks of age, and then subjected for behavioral examinations before brain sample collections at 9 weeks of age (Fig. 5A, bottom panel). We first compared the body weight at 9 weeks of age, and results show that there is no difference between these two groups (Fig. 5B). We further examined the motor functions via rotarod and grip strength tests. As shown in Fig. 5C and D, GIGF2-injected mice display significantly longer retention time on the rotarod compared to that of control mice (Fig. 5C); however, these two groups do not show significant difference for the grip strength (Fig. 5D), suggesting IGF2 partially improves motor functions. To further analyze the neuropathological mHTT aggregates, we performed immunostaining and Western blotting using an EM48 antibody. For the results of immunostaining, since both the lentiviral vectors carry the *GFP* reporter genes, the infected cells with GFP signals could be observed in striatum (Fig. 5E, top panel). In the GIGF2 group, significantly less mHTT signals are observed compared to that of control group (Fig. 5E middle panel; Fig. 5F). We further subjected striatum samples from two groups for Western blotting (Fig. 5G), showing GIGF2 group has higher level of exogenous IGF2 and displays significantly less mHTT in stacking gel (Fig. 5G and H). Moreover, p-AKT/T-AKT ratio is determined by Western blotting as well (Fig. 5G), revealing that GIGF2 slightly increases p-AKT/T-AKT ratio compared to that of the control group (Fig. 5I). Taken these results together, IGF2 offers beneficial functions to improve motor functions, to decrease mHTT aggregates and to slightly increase p-AKT/T-AKT ratio in R6/2 HD transgenic mice in vivo.

## Discussion

The accumulation of mHTT is a critical pathological characteristic, leading to cellular dysfunctions and ultimately cell death in HD. As a result, clearance of mHTT is one of directions to provide therapeutical treatments in HD. In this study, we provide evidence that IGF2 reduces mHTT aggregates by activating the AKT/NF-κB signaling to promote the secretion of mHTT in extracellular vesicles. Since previous studies have shown IGF2 is beneficial for several neurodegenerative diseases, which all display aggregates of disease-causing proteins [17–19], our study may provide an insight of advanced mechanisms toward the neuroprotective effects of IGF2 in these diseases.

Our laboratory has shown IGF2 enhances neuronal cytoskeletons to protect neurons in HD models [9]. In this study, we further have demonstrated the IGF2 suppresses the mHTT aggregates through AKT/NF-κB signaling. These highly suggest IGF2 could be an important target to provide neuroprotective effects, which are also supported by other studies. For example, IGF2 has been shown to reduce amyloidosis, stimulate neurogenesis and synaptogenesis, further improving cognitive functions in AD [17, 41]. Moreover, IGF2 also has reported to increase dendritic spines and dopaminergic neurons and to reduce cytotoxicity and apoptosis triggered by alpha-synuclein intracellular accumulation in PD mouse models [18]. These results definitely support the neuroprotective roles of IGF2 in different neuronal diseases, but imply the complicated working mechanisms of IGF2 as well. Especially, we found IGF2 treatment increases mHTT mRNA (Supplementary Fig. 2), but also observed IGF2 decreases mHTT aggregates(Fig. 1C and D). Since it has been reported that IGF2 binds to its receptors to activate different intracellular signaling pathways, such as PI3K/AKT and MAPK/ERK, and activate key transcription factors (e.g., NF-κB, CREB, c-Myc) or influence epigenetic modifications and chromatin accessibility [15, 42], it would be important to further demonstrate the advanced regulatory mechanisms of IGF2 if this innovative treatment will be applied.

In this study, we show the activation of AKT/NF-κB signaling is a critical for IGF2 functions in HD. Based on previous studies, AKT not only regulates different cellular functions, such as cell survival, proliferation, glucose uptake, angiogenesis, and metabolism, etc [21, 22]., but also provides neuroprotective effects through mTOR and GSK3β downstream signaling [23–25], highly suggesting IGF2 may also partially reveal above characteristics in our models. It could be expected that the treatment of IGF2 may also lead to certain side-effects. As a result, although we provide proof of concept for IGF2 beneficial effects in this study, longitudinal examinations and optimization of treatments should be addressed.

Another important downstream of IGF2 is NF-κB signaling in this study (Fig. 3). Due to that NF-kB may be associated with cellular EV production [37, 38], we link IGF2 activates AKT to further lead to NF-κB signaling. Although we prove IGF2 activates NF-κB to increase secreted mHTT in EVs of culture media (Figs. 3 and 4), the double-edged role of NF-κB should be considered. NF-kB is a well-known transcription factor that regulates the inflammatory cascades to cause oxidative stress and neurodegeneration [29, 36]. However, activation of NF-κB signaling has also been reported to provide neuroprotective effects [28]. These results suggest NF-κB signaling may play different positive or negative roles under different conditions, leading to neuroprotective or neurodegenerative effects in the neuronal system. Due to the dual roles of NF-κB signaling, the pros and cons of IGF2 should also be considered, particularly in relation to modulating NF-κB activity to optimize EV secretion while controlling inflammation. In addition, although we observed IGF2 significantly increases EV production, which contains more mHTT aggregates (Fig. 4), we do not have evidence to show this EV production is the major or minor player to reduce mHTT aggregates. IGF2 has been reported to activate autophagy, which is one of critical protein degradation pathways, in other disease models [43, 44], suggesting IGF2 may also remove mHTTs through the autophagy pathway. As a result, further understanding the multiple effects of IGF2 on clearance of mHTT aggregates would be an important topic if the therapeutical target of IGF2 would be applied in HD.

In our in vivo models, we show stereotactic injection of IGF2 not only improves rotarod behavior and decreases mHTT aggregates, but also slightly increases level of p-AKT/AKT ratio in R6/2 HD transgenic mice (Fig. 5). However, we do not observe the improvement of body weight and grip strength after the IGF2 treatments in vivo. Since stereotactic injection of exogenous viruses can only infect a small, specific area of brain cells, the limited delivery of foreign IGF2 may result in restricted therapeutic effects. To examine this issue, germ-line IGF2 transgenic models could be used. For example, we could generate IGF2 transgenic mice driven by a neuron specific promoter, such as a prion promoter, to overexpress IGF2 in all neurons, and then breed with HD transgenic mice to generate IGF2-HD double transgenic mice. With the broad distribution of IGF2, the better improvement of phenotypes may be observed. Moreover, another issue in vivo is that IGF2 only slightly induces activation of the AKT signaling (Fig. 5I), which is not consistent to the in vitro data (Fig. 2). As mentioned previously, AKT signaling regulates several different cellular functions [21, 22]. Therefore, AKT signaling may be activated at different conditions, suggesting the timing of tissue sampling after IGF2 injection may be a critical factor. As a result, the more significant increase of p-AKT/AKT ratio may be observed if we collected brain samples at earlier stage.

## Conclusions

To sum, this study demonstrates that IGF2 reduces mHTT aggregates in HD by activating the AKT/NF-κB signaling pathway, leading to increased mHTT secretion via EVs. In HD transgenic mice, IGF2 administration improved motor function, reduced mHTT aggregates, and modestly elevated AKT phosphorylation. These findings underscore IGF2’s therapeutic potential in HD by promoting mHTT clearance through AKT/NF-κB-mediated pathways.

## Electronic supplementary material

Below is the link to the electronic supplementary material.

Supplementary Material 1

Supplementary Material 2

## Data Availability

All data supporting the findings of this study are available within the paper and its Supplementary Information.

## Abbreviations

mHTT: mutant Huntingtin
HD: Huntington’s disease
IGF2: Insulin-like growth factor 2
AKT: Protein Kinase B
NF-κB: nuclear factor kappa-light-chain-enhancer of activated B cells
IGF: insulin-like growth factor
IGF2R: insulin-like growth factor 2 receptor
CNS: central nervous system
mTOR: mammalian target of rapamycin
GSK3β: glycogen synthase kinase 3 beta
IKK: IκB kinase
EGFP: enhanced green fluorescent protein
CMV: cytomegalovirus
SDS-PAGE: SDS polyacrylamide gel electrophoresis
PVDF: polyvinylidene difluoride
PCR: polymerase chain reaction
IACUC: Institutional Animal Care and Use Committee
GEO: Gene Expression Omnibus
p-AKT: phosphor-AKT
T-AKT: total AKT
shRNA: short hairpin RNA
EVs: extracellular vesicles
