# Supplementary material for: Insulin-like growth factor 2 reduces Huntington’s disease aggregates via AKT and NF-κB signaling in huntington’s disease
Source: Cell Biosci. 2025 Jul 26;15:109. doi: 10.1186/s13578-025-01452-4 (PMC12297735; doi:10.1186/s13578-025-01452-4)
Supplement: Supplementary file 1 — Supplementary Material 1 [file 13578_2025_1452_MOESM1_ESM.docx]

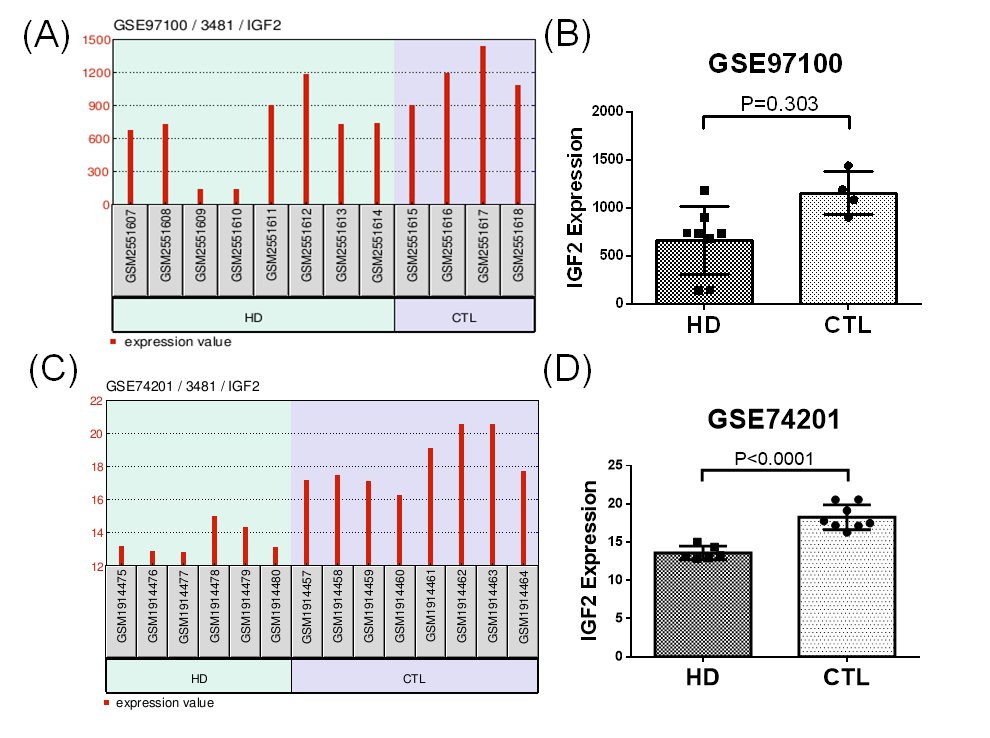


**Supplementary Figure 1. Human HD models show lower IGF2 expression level. (A and B)** IGF2 gene expression in HD patient iPSC-derived brain microvascular endothelial cells (N=8) and control(CTL) cells (N=4) obtained from the GEO database (GSE97100). **(A)** Raw data of IGF2 mRNA expression in iPSC-derived brain microvascular endothelial cells. **(B)** Quantitative analysis from (A) shows a significant decrease of IGF2 in HD cells. **(C and D)** IGF2 gene expression in HD patient iPSC-derived differentiated neural stem cells (N=6) and control cells (N=8) obtained from the GEO database (GSE74201). **(C)** Raw data of IGF2 mRNA expression in iPSC-derived differentiated neural stem cells. **(D)** Quantitative analysis from (C) shows a significant decrease of IGF2 in HD cells.

**Supplementary Figure 2. IGF2 induces the mRNA levels of mHTT in N2a cells.** N2a cells were transfected mHTT, G84Q, with control(Cont.) empty vector or IGF2 for 48 hours, and then subjected for real-time quantitative PCR. Results show IGF2 induces mHTT mRNA expression in HD cells. Values represent the mean ± SD. ***p < 0.001.


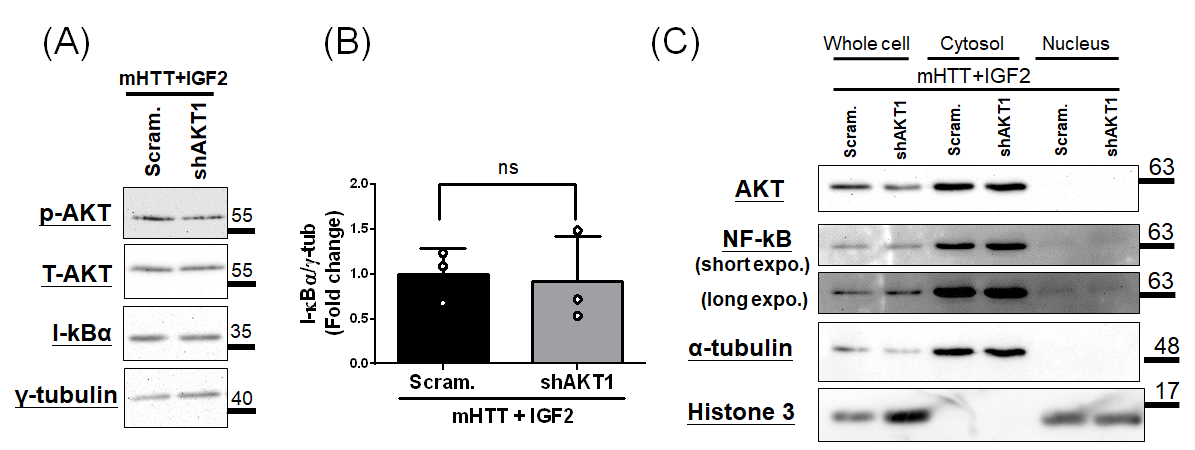


**Supplementary Figure 3. The NF-κB is not translocated into nucleus after the IGF2 treatment in HD cells as AKT1 is knocked down.** N2a cells were transfected mHTT and IGF2 with scramble shRNA(Scram.) or shAKT1 for 48 hours, and then subjected for Western blotting and nuclear and cytoplasmic fractionation. **(A)** Western blotting shows the expression of p-AKT, AKT, I-κBα after transfection of different constructs. γ-tubulin serves as an internal control. **(B)** Quantitative analysis from (A) shows a similar level of I-κBα level after the shAKT1 treatment. **(C)** Western blotting from nuclear and cytoplasmic fractionation shows the similar expression of NF-kB after shAKT1 treatment in nucleus. Histone 3 and α-tubulin were used as indicators for nuclear and cytosolic fractions, respectively. Data represent the mean ± SD.


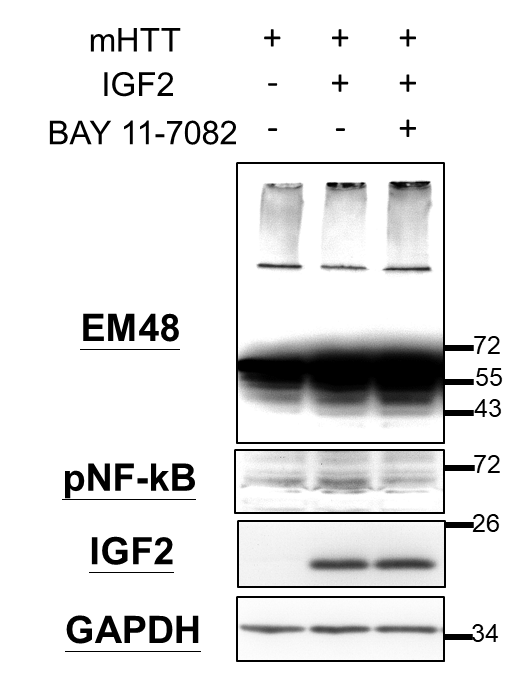


**Supplementary Figure 4. BAY 11-7082, an NF-κB Inhibitor, reverses the suppression of mHTT aggregates induced by IGF2.** N2a cells were pretreated with or without BAY 11-7082 for 1 hours before transfection. The pretreated cells were transfected mHTT with or without IGF2 for 48 hours, and then subjected for Western blotting. Western blotting shows the expression of EM48, pNF-kB and IGF2 after different treatments. GAPDH serves as an internal control.
