# Supplementary material for: Insulin-like growth factor 2 reduces Huntington’s disease aggregates via AKT and NF-κB signaling in huntington’s disease
Source: Cell Biosci. 2025 Jul 26;15:109. doi: 10.1186/s13578-025-01452-4 (PMC12297735; doi:10.1186/s13578-025-01452-4)

Figure 1E

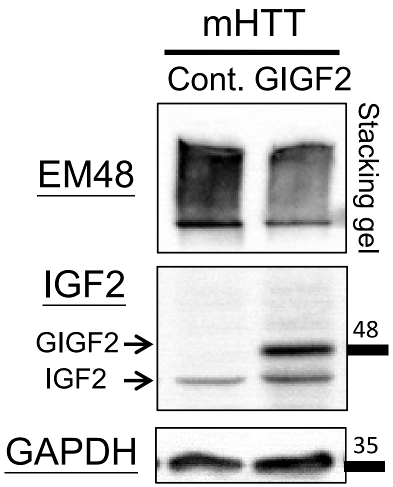

mHTT
Cont. GIGF2
EM48
Stacking gel
IGF2
GIGF2
IGF2
48
GAPDH
35

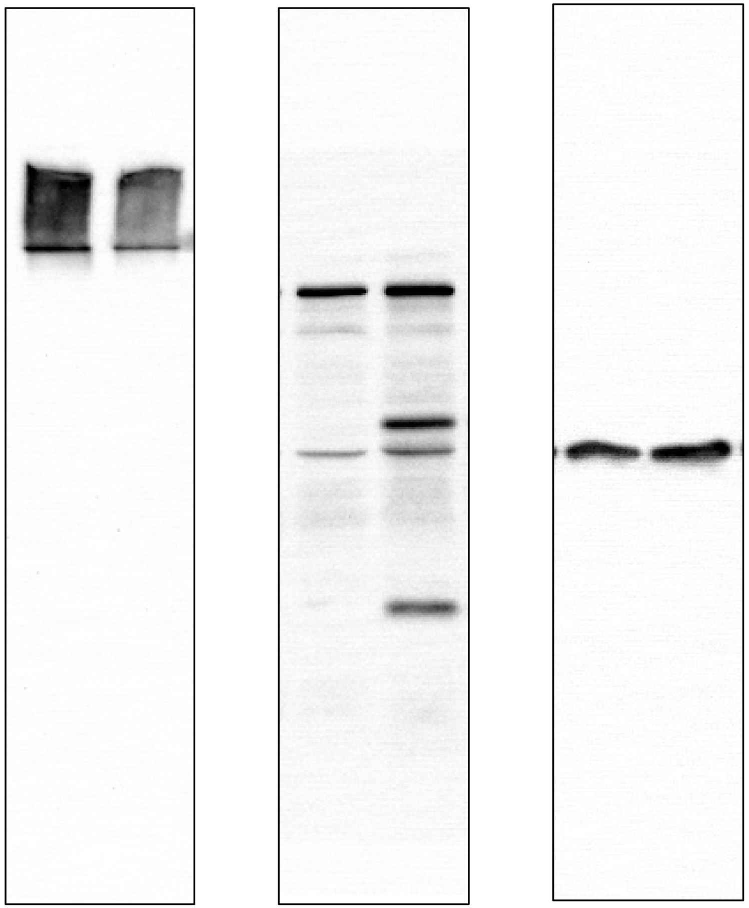

Figure 2A

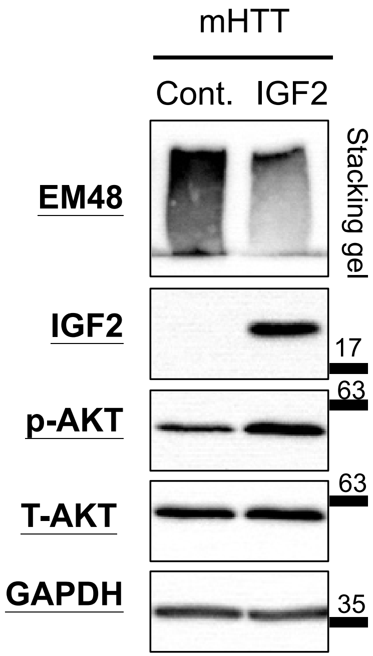

mHTT
Cont.
IGF2
EM48
Stacking gel
IGF2
17
p-AKT
63
T-AKT
63
GAPDH
35

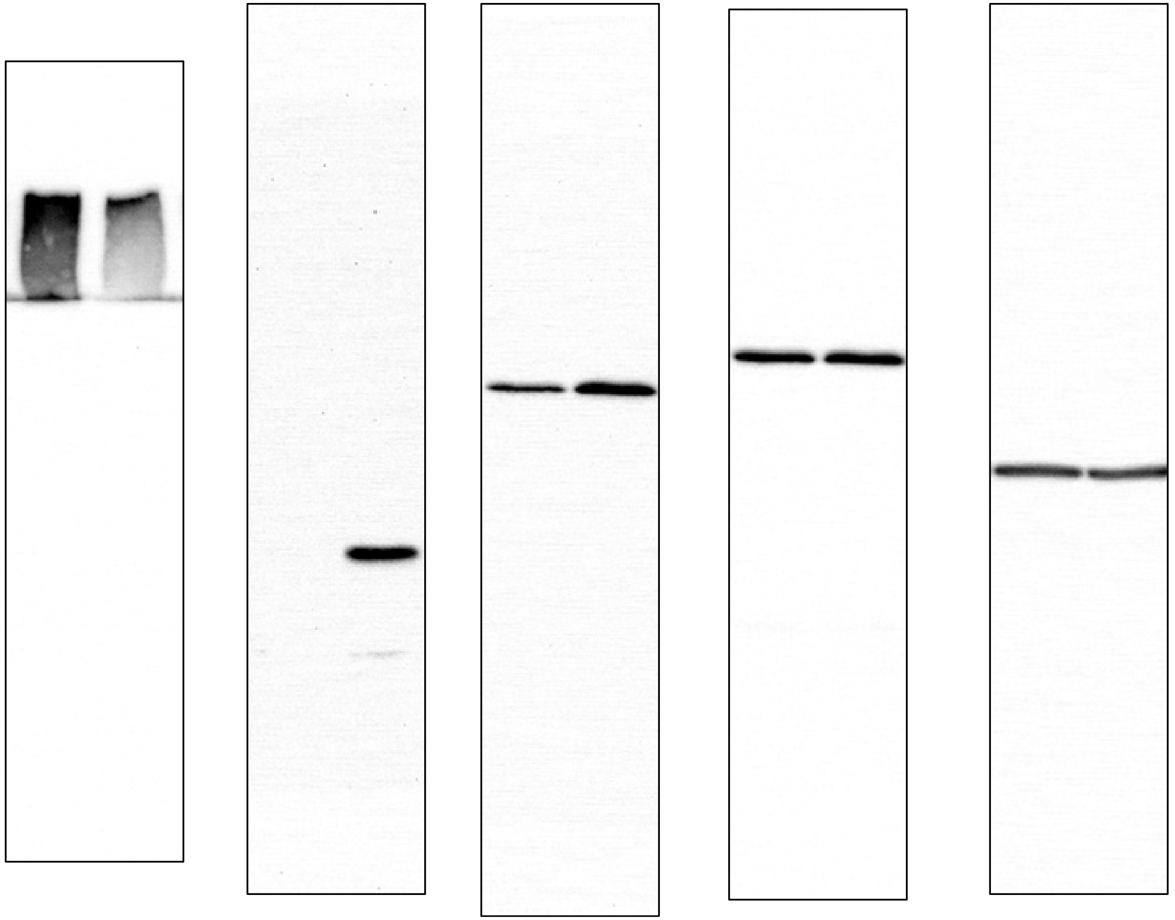

Figure 2C

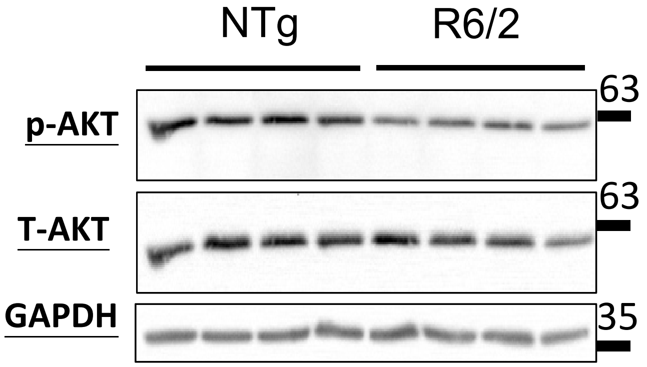

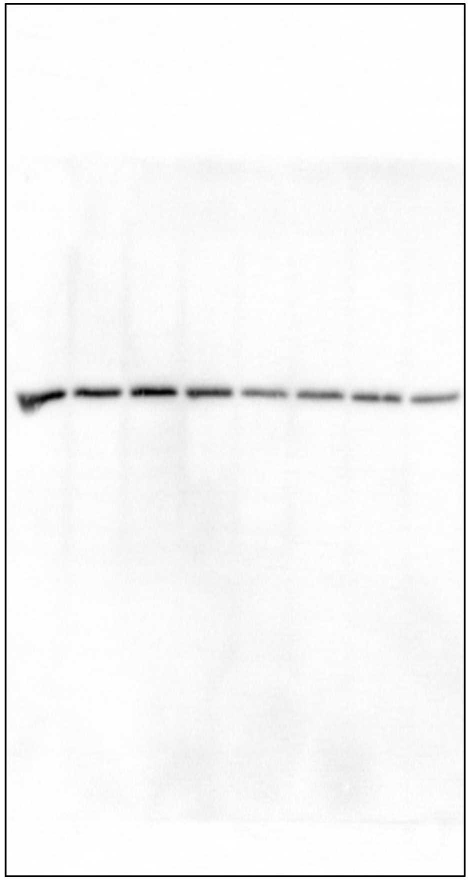

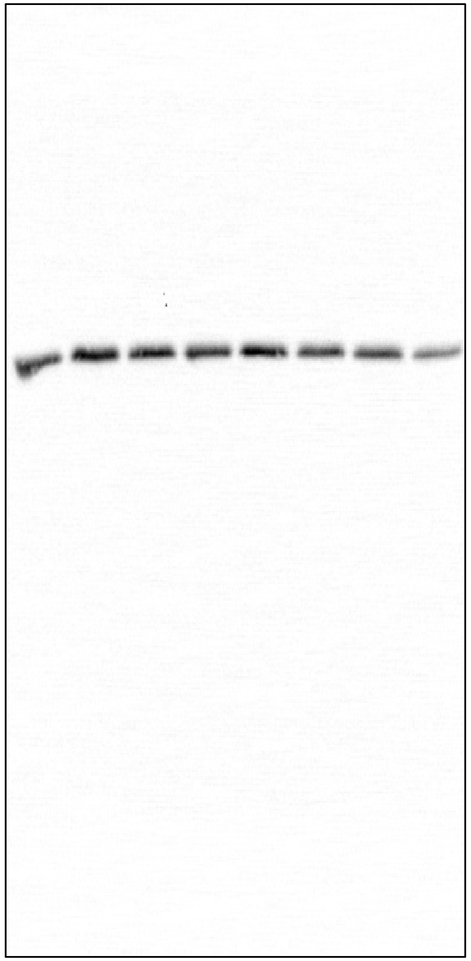

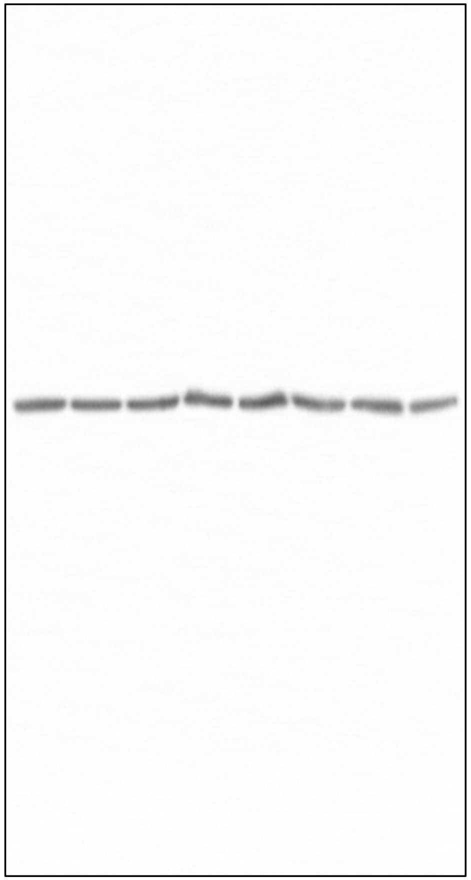

Figure 2G

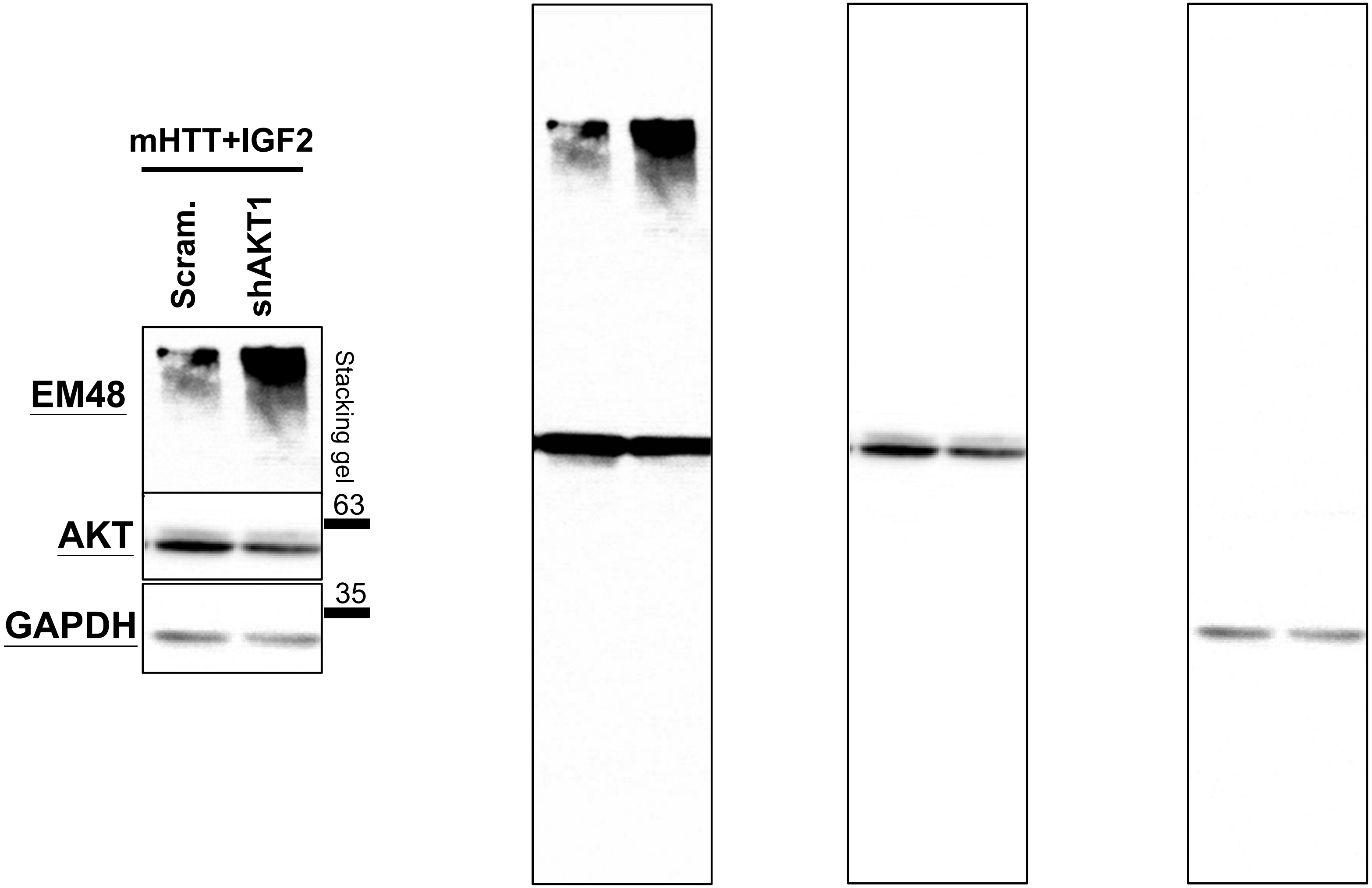

Figure 3A

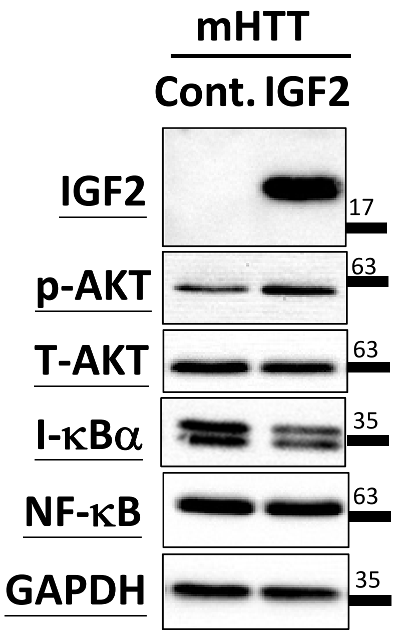

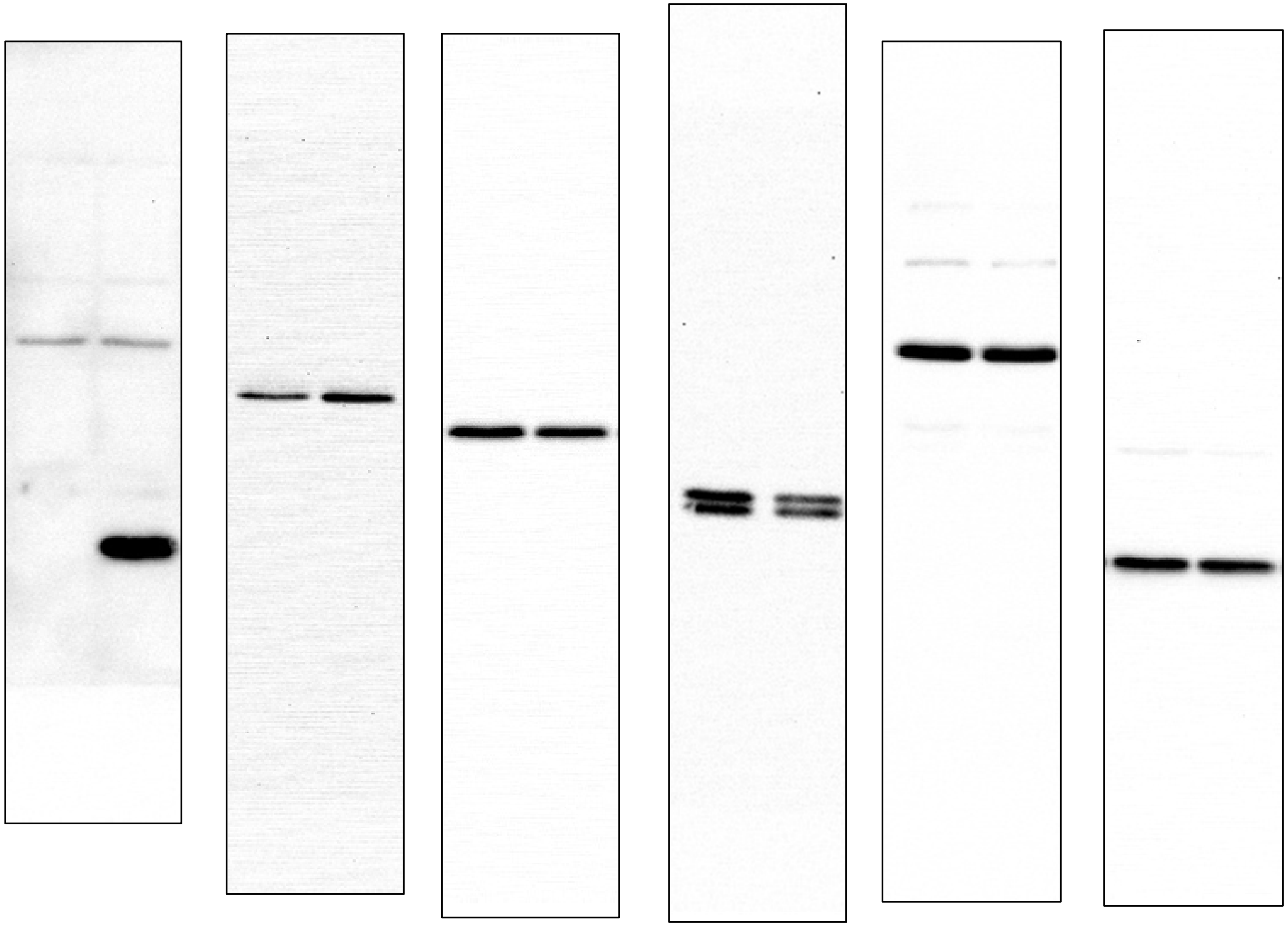

Figure 3C-1

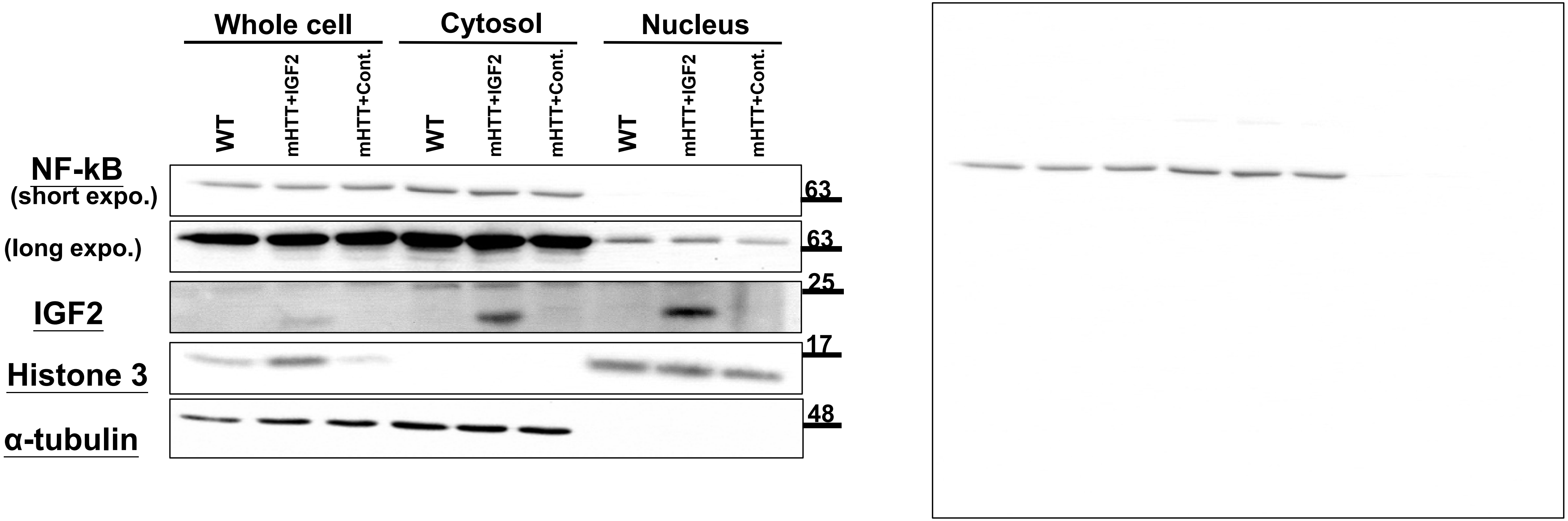
Whole cell
Cytosol
Nucleus
WT
mHTT+IGF2
mHTT+Cont.
WT
mHTT+IGF2
mHTT+Cont.
WT
mHTT+IGF2
mHTT+Cont.
NF-kB
(short expo.)
(long expo.)
IGF2
Histone 3
α-tubulin
63
63
25
17
48

Figure 3C-2

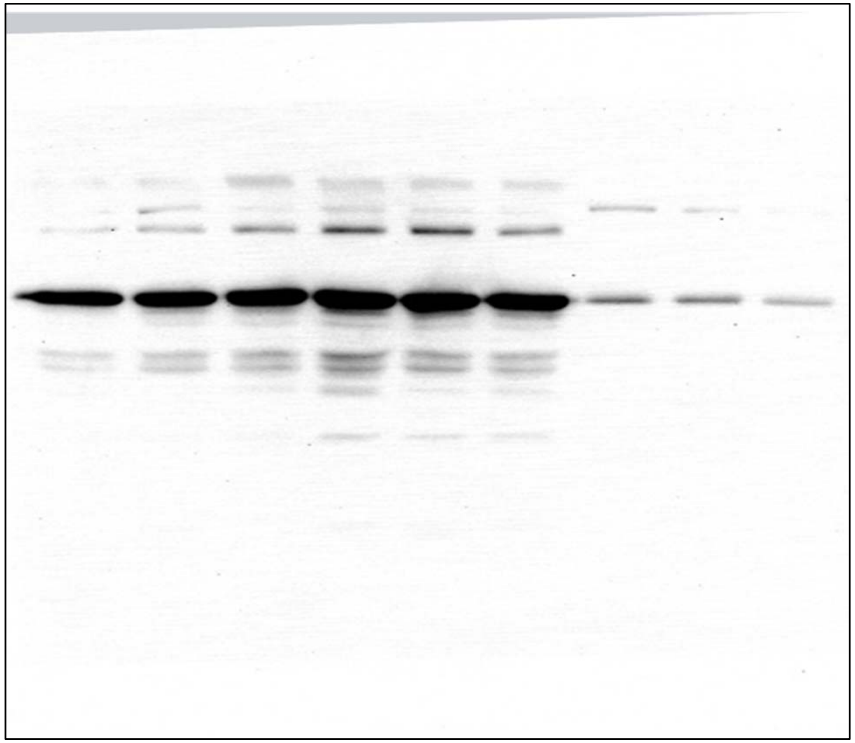

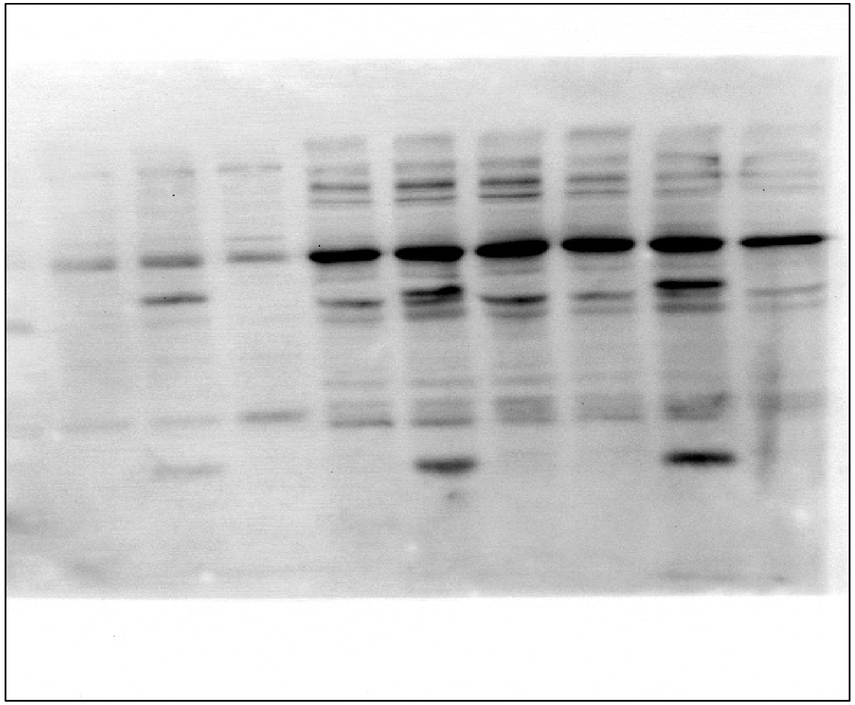

Figure 3C-3

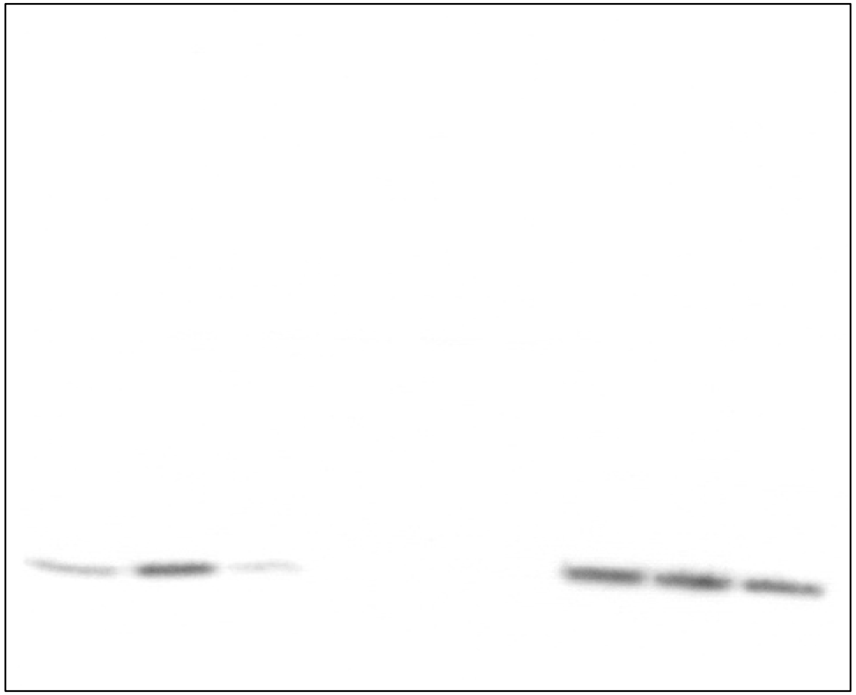

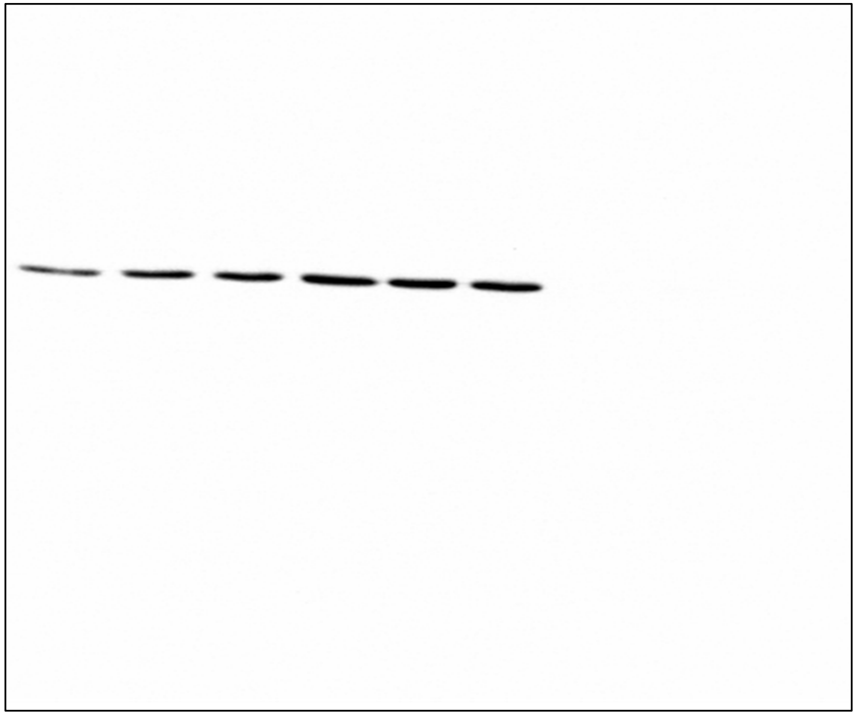

Figure 4A

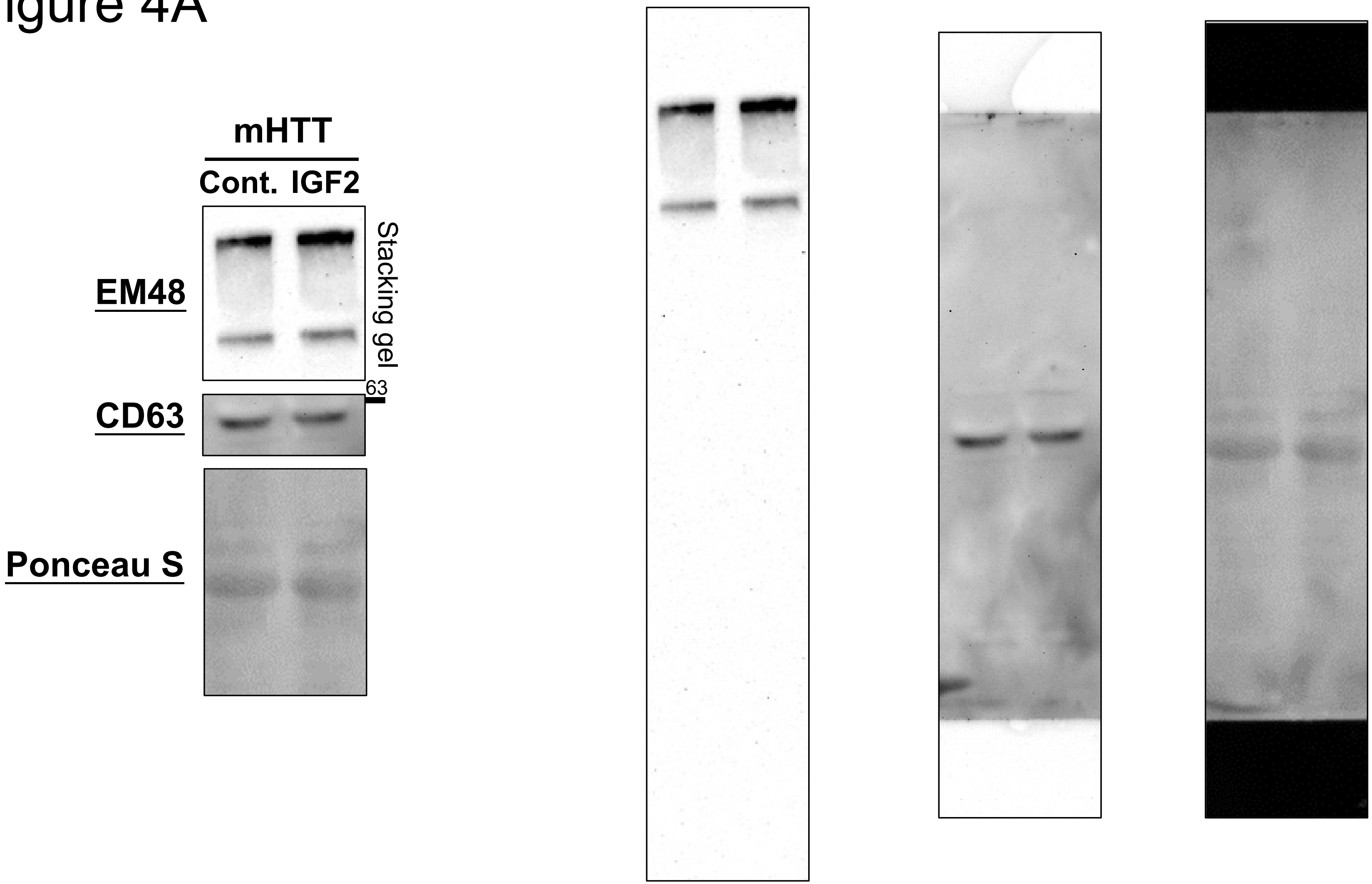

Figure 4C

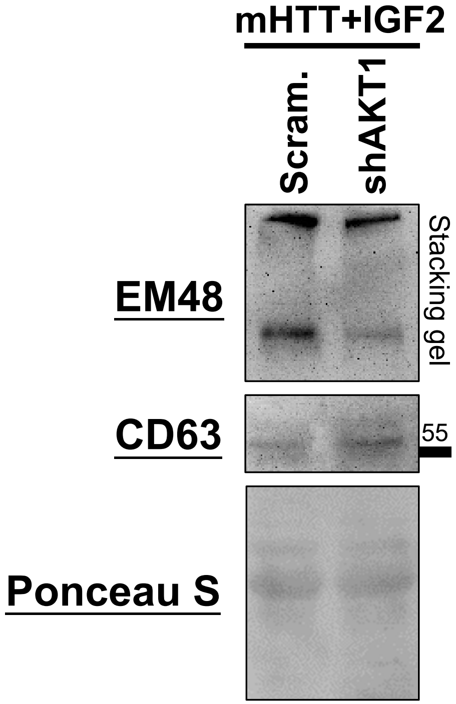

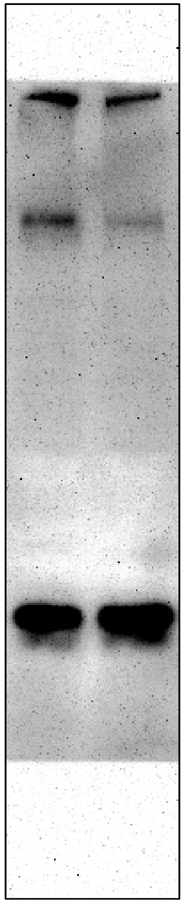

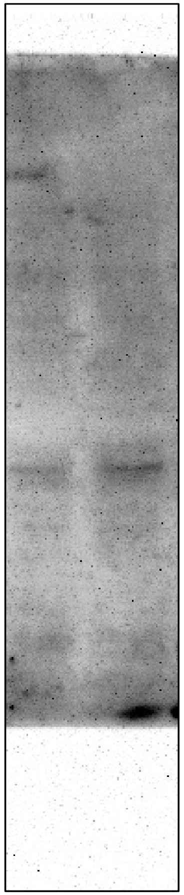

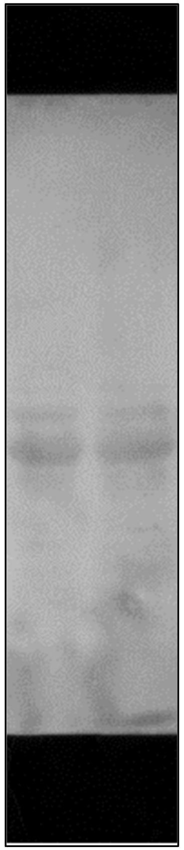

Figure 5G

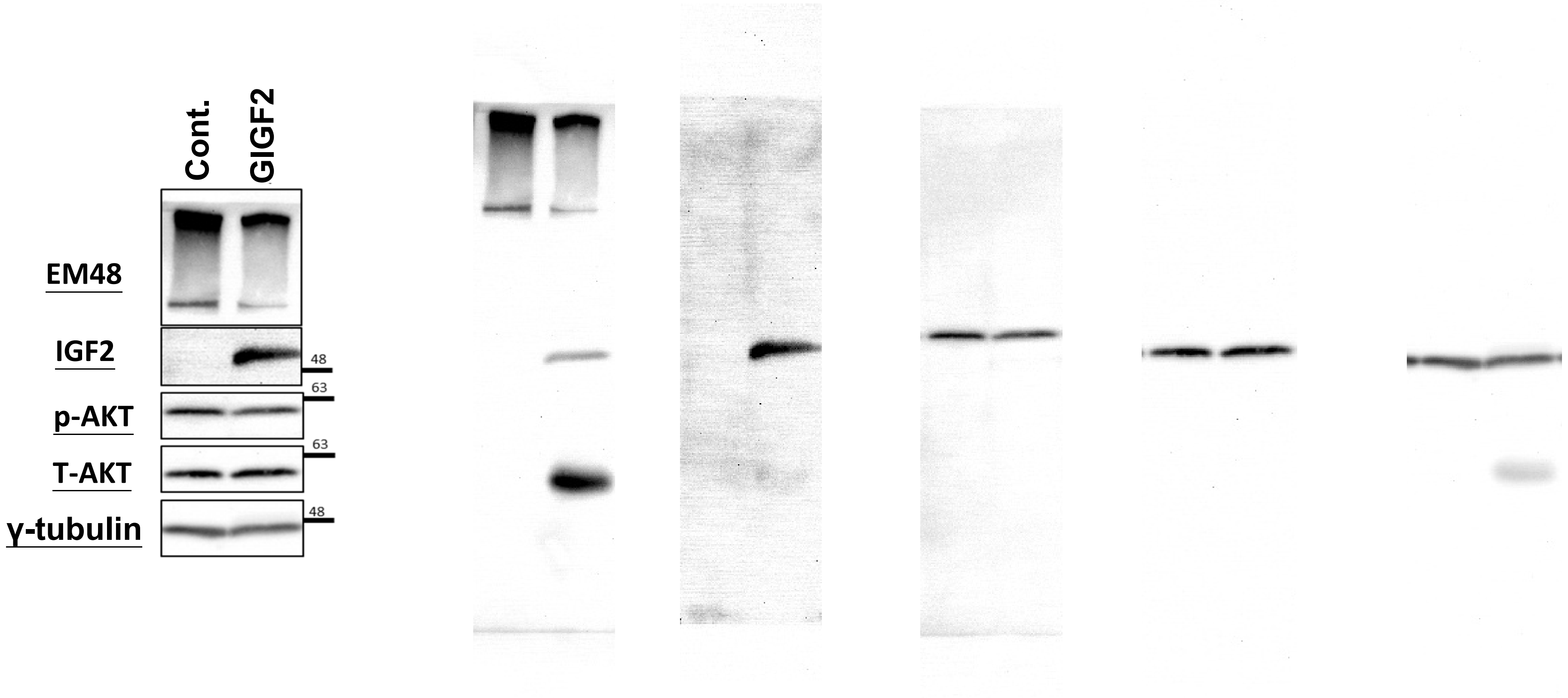

Supplementary Figure 3A

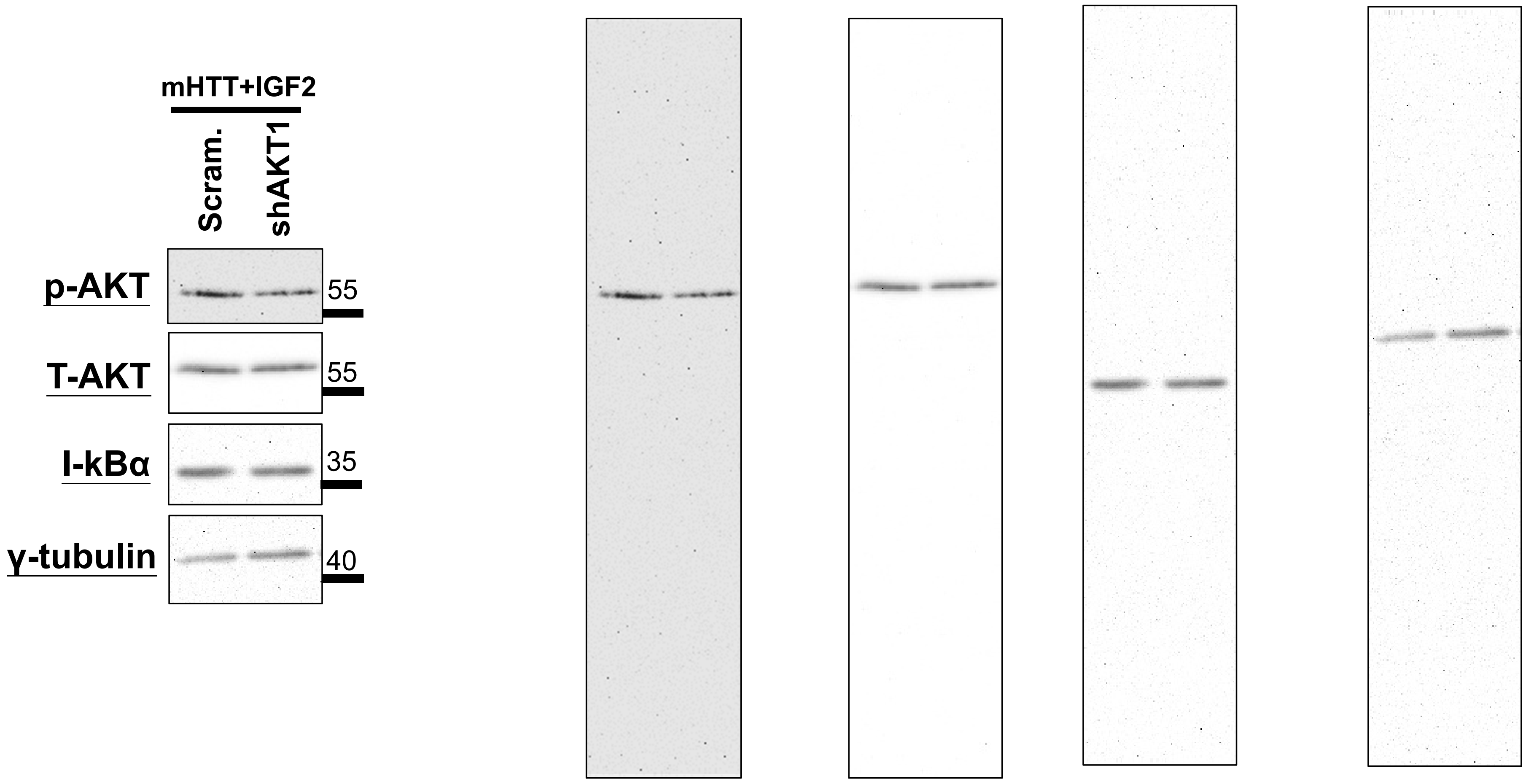

Supplementary Figure 3C-1

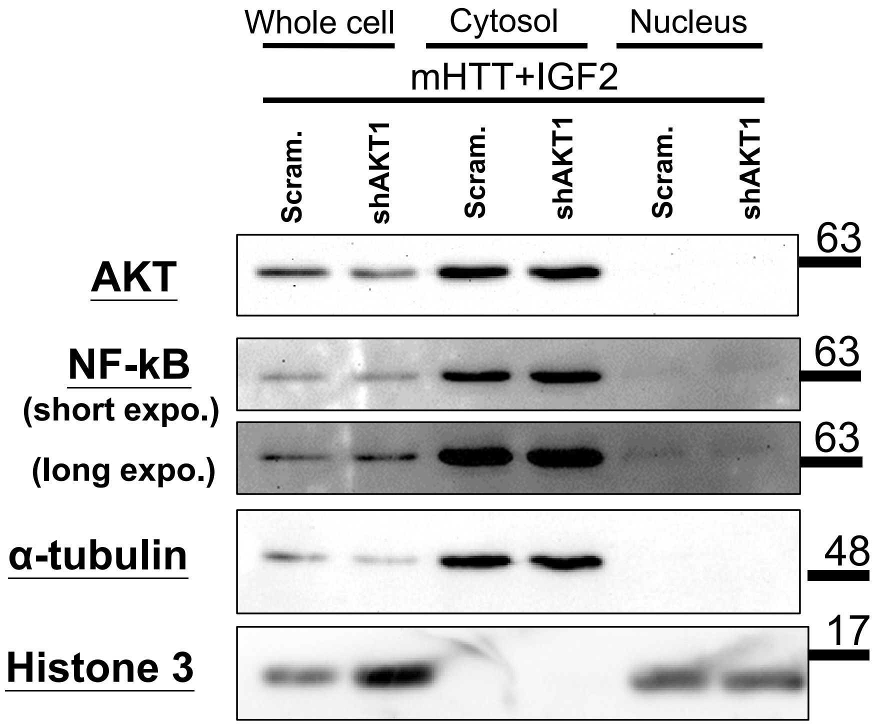

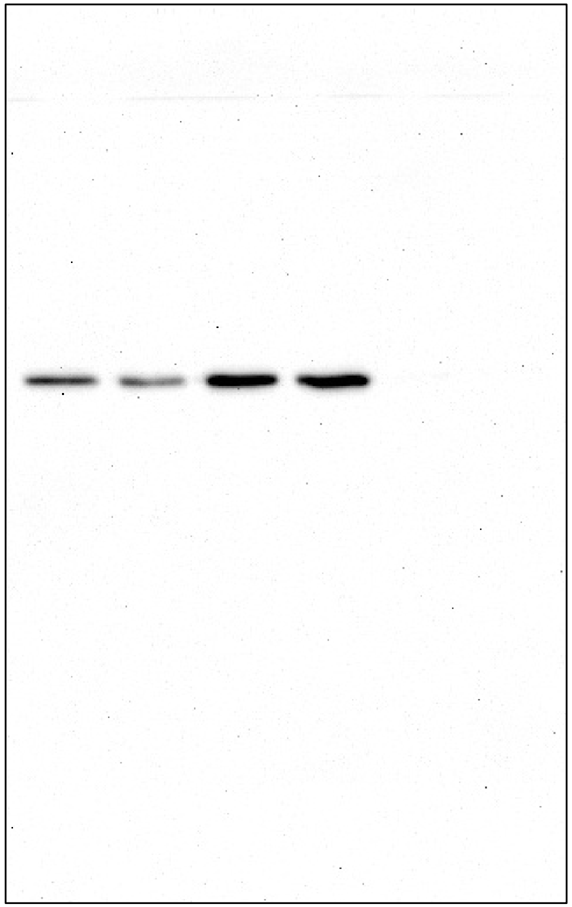

Supplementary Figure 3C-2

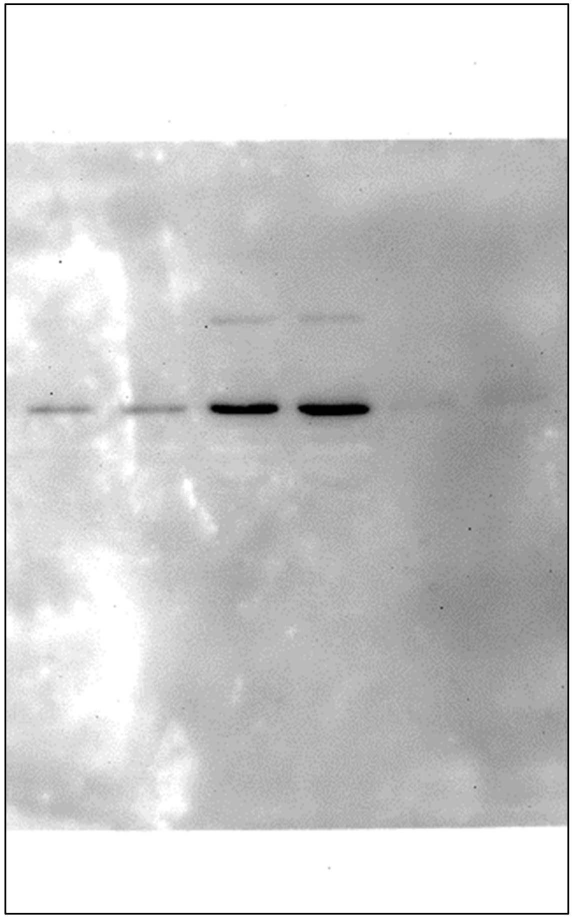

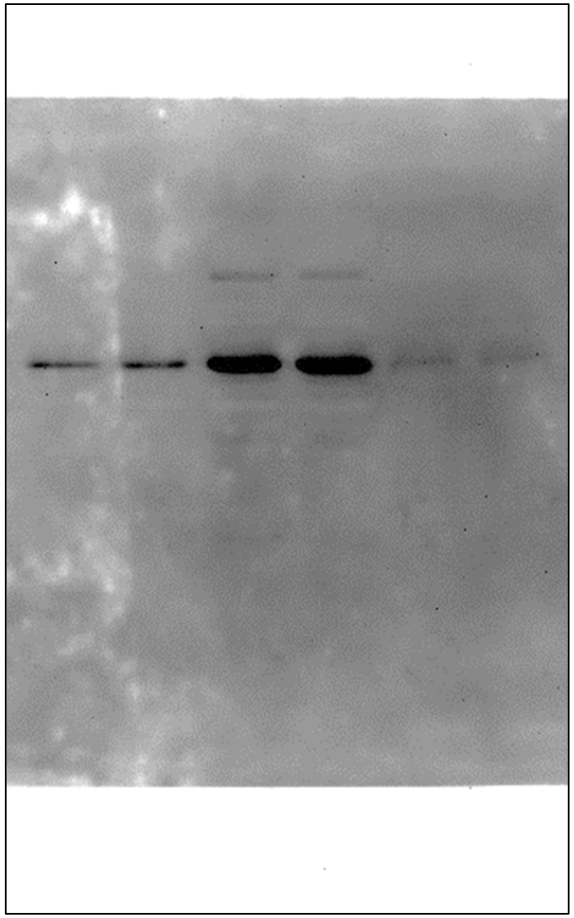

Supplementary Figure 3C-3

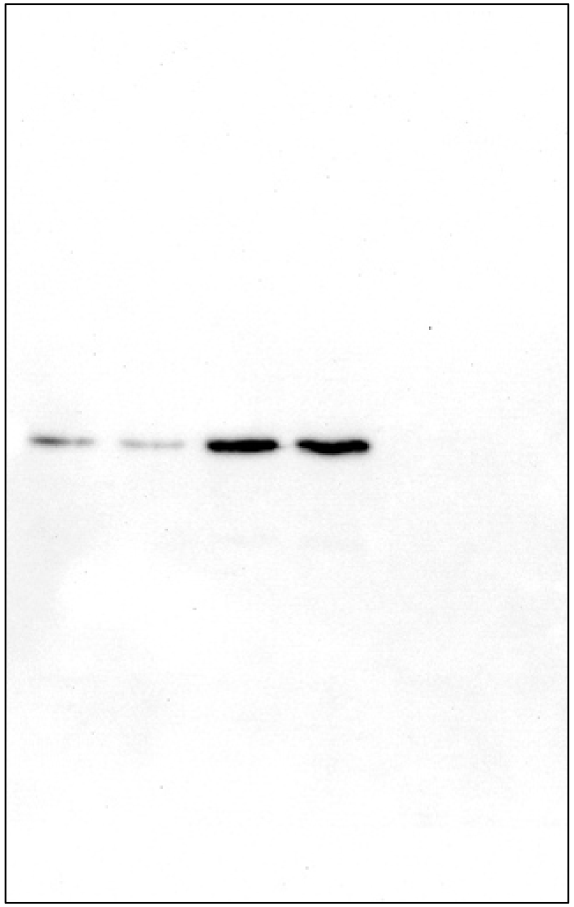

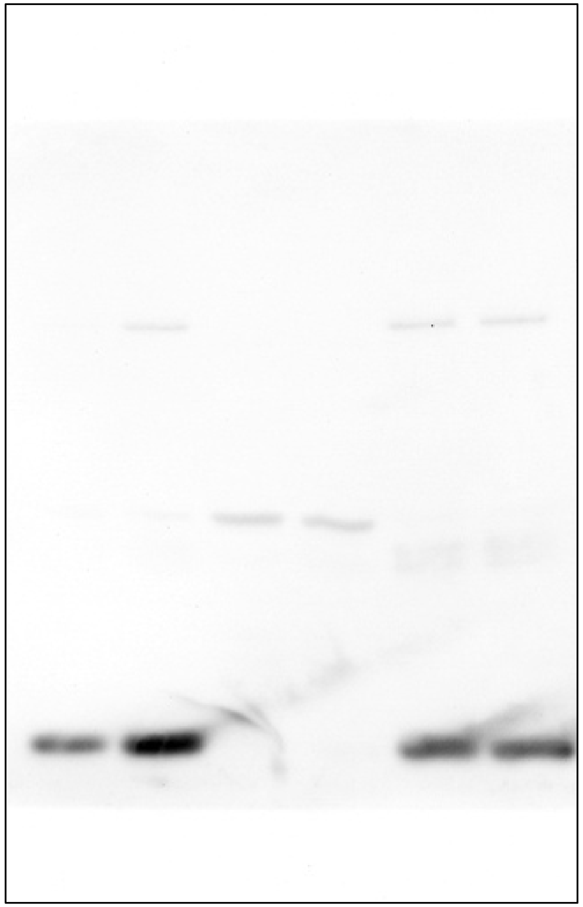

Supplementary Figure 4

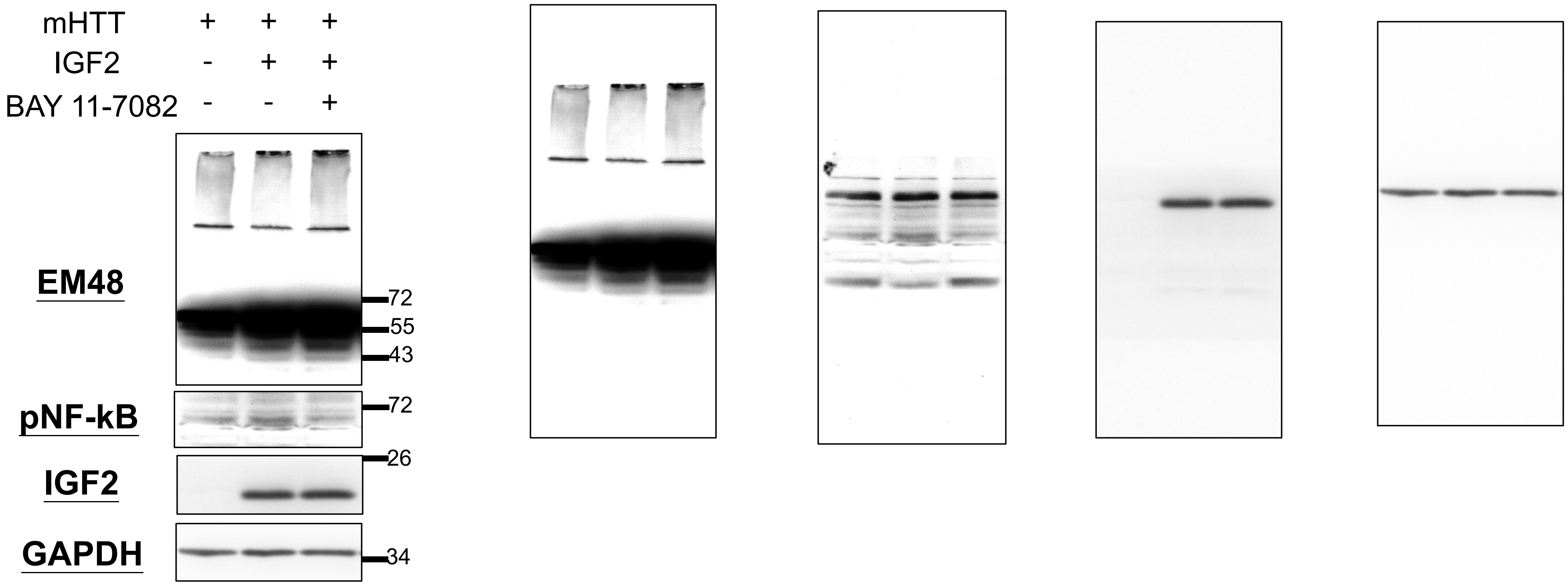

Supplement: Supplementary file 2 — Supplementary Material 2 [file 13578_2025_1452_MOESM2_ESM.pdf]
